# Pre-surgery immune profiles of adult glioma patients

**DOI:** 10.1007/s11060-022-04047-y

**Published:** 2022-06-18

**Authors:** Paige M. Bracci, Terri Rice, Helen M. Hansen, Stephen S. Francis, Sean Lee, Lucie S. McCoy, Pavan P. Shrestha, Gayathri Warrier, Jennifer L. Clarke, Annette M. Molinaro, Jennie W. Taylor, John K. Wiencke, Margaret R. Wrensch

**Affiliations:** 1 Department of Epidemiology and Biostatistics, UCSF, 1450 3rd Street, San Francisco, CA 94158 USA; 2 Department of Neurological Surgery, UCSF, San Francisco, CA USA; 3 Department of Neurology, UCSF, San Francisco, CA USA

**Keywords:** Glioblastoma, Lower grade glioma, Dexamethasone, Immune cell subset, Methylation, Deconvolution methods

## Abstract

**Introduction:**

Although immunosuppression is a known characteristic of glioma, no previous large studies have reported peripheral blood immune cell profiles prior to patient surgery and chemoradiation. This report describes blood immune cell characteristics and associated variables prior to surgery among typical glioma patients seen at a large University practice.

**Methods:**

We analyzed pre-surgery blood samples from 139 glioma patients diagnosed with a new or recurrent grade II/III glioma (LrGG, n = 64) or new glioblastoma (GBM, n = 75) and 454 control participants without glioma. Relative cell fractions of CD4, CD8, B-cells, Natural Killer cells, monocytes, and neutrophils, were estimated via a validated deconvolution algorithm from blood DNA methylation measures from Illumina EPIC arrays.

**Results:**

Dexamethasone use at time of blood draw varied by glioma type being highest among patients with IDH wild-type (wt) GBM (75%) and lowest for those with oligodendroglioma (14%). Compared to controls, glioma patients showed statistically significant lower cell fractions for all immune cell subsets except for neutrophils which were higher (all p-values < 0.001), in part because of the higher prevalence of dexamethasone use at time of blood draw for IDHwt GBM. Patients who were taking dexamethasone were more likely to have a low CD4 count (< 200, < 500), increased neutrophils, low absolute lymphocyte counts, higher total cell count and higher NLR.

**Conclusion:**

We show that pre-surgery blood immune profiles vary by glioma subtype, age, and more critically, by use of dexamethasone. Our results highlight the importance of considering dexamethasone exposures in all studies of immune profiles and of obtaining immune measures prior to use of dexamethasone, if possible.

**Supplementary Information:**

The online version contains supplementary material available at 10.1007/s11060-022-04047-y.

## Introduction

Although immunosuppression is a well-known feature of glioma [1, 2], no previous large studies of peripheral blood immune cell profiles prior to surgery and chemoradiation have been reported. We previously showed that post-diagnosis immune profiles of glioma patients may be helpful in predicting survival [3, 4]. In a recent study using survival models that included clinical factors and immune cell profiles, we showed that patients > 58 years old with high neutrophil proportions had the poorest survival, whereas for patients  ≤58 years old, low CD4 T cell proportions were associated with poor survival [4]. These prior studies relied on a single post-diagnosis blood sample. Given this limitation we designed an Immune Profile Study (IPS) to obtain blood samples and analyze longitudinal immune characteristics at clinically relevant time points for glioma patients. This report from the UCSF IPS examines distributions of immune cell types and ratios in blood samples obtained before surgery from glioma patients stratified by tumor WHO 2016 type, recurrence status [newly diagnosed or recurrent lower grade glioma (LrGG)], and other potentially relevant patient characteristics including dexamethasone use at time of blood draw based on its known systemic immunosuppressive effects and use across the treatment spectrum for many patients with GBM.

## Methods

### Study participants and blood samples

#### Glioma patients

Pre-surgery blood samples were collected from newly diagnosed glioblastoma (GBM) or LrGG or recurrent LrGG patients as part of the IPS. All patients who were able to provide informed consent, resided in the US, spoke English and were scheduled for a biopsy or resection at UCSF between February 23, 2018 and November 20, 2019 for a presumed new glioma or recurrence of a LrGG were eligible. Patient’s pathologic diagnosis was abstracted from medical records. Patients in this study were diagnosed prior to implementation of the WHO 2021 revised classification of adult glioma and the neuropathology review to recategorize them from the WHO 2016 to WHO 2021 classification is not yet completed. However, the WHO 2016 categories used here generally correspond as follows: WHO 2016 IDHwt GBM is equivalent to that category in WHO 2021, WHO 2016 IDHmt GBM is equivalent to WHO 2021 IDHmt astrocytoma grade 4, IDHmt astrocytoma is equivalent to WHO IDHmt astrocytoma grade 2, 3, and WHO 2016 IDHmt 1p/19q co-deleted oligodendroglioma is equivalent to that category in the WHO 2021.

Informed consent was obtained from each study participant.

#### Control participants

Blood samples from people who did not have glioma were collected by the Adult Glioma Study (AGS) between 1991 and 2012 at the time of enrollment [5]. These controls were identified through random digit dialing in the San Francisco Bay Area (1991–1994, 1997–1999) or from a UCSF Phlebotomy Clinic (2006–2012). All participants provided informed consent. This study included 454 such controls from previous studies with blood methylation data [3, 4].

Glioma patient pre-surgery blood samples were typically obtained the day prior to surgery; none were obtained during or after exposure to anesthesia. Samples were processed within 48 h of collection. A pre-surgery blood draw questionnaire given to the patient, asked about daily and cumulative dexamethasone exposure. Additional details were abstracted from medical records. For pre-surgery samples, total blood nucleated cell counts were measured by Nexcelcom cell cytometry (Lawrence, MA USA) before freezing with 60 μl citrated EDTA anticoagulated whole blood. Cell counts were not available for control participants. This study was approved by the University of California, San Francisco IRB.

### DNA methylation array

Frozen (− 80 °C) anticoagulated whole blood or isolated cells were processed, DNA isolated, and bisulfite converted as previously described [6]. All samples and array experiments were performed blinded to clinicopathologic variables. Approximately 200–500 ng of DNA was applied to Infinium Methylation EPIC BeadChip arrays. Preprocessing and quality control of fluorescence data were accomplished in R v1.4 [7] using the minfi Bioconductor package [8]. To ensure high-quality methylation data, CpG loci having a sizable fraction (> 25%) of detection p values above a predetermined threshold were filtered (detection p > 10E^−5^) [9]. A ‘noob’ background correction method was used to account for background fluorescence and dye bias [10]. Beta-mixture quantile normalization was performed to correct for probe design bias [11]. The presence of technical sources of variability induced by plate and/or BeadChip was examined using principal components analysis, and the top K principal components [12] were examined in terms of their association with plate and BeadChip. The final data set contained 830,277 probes.

### Immunomethylomic analysis

Our previously published immunomethylomic deconvolution method provides the proportions of six major cell types (CD4 T, CD8 T, B-cell, Natural Killer (NK), monocytes, neutrophils) and the neutrophil/lymphocyte (NLR), CD4/CD8, and lymphocyte/monocyte (LMR) ratios. DNA methylation-based immune profiles are highly accurate and reproducible [6, 13, 14]. Specifically, the proportions of each cell were estimated for each sample using the function “estimateCellCounts2” in the FlowSorted Blood EPIC Bioconductor package. Ratios of cell types were estimated as previously described [15]. Absolute cell counts for cell types were obtained by multiplying cell type proportions by each sample’s total white cell count. Only cell proportions were estimated for the historical AGS controls as total cell count data were not available.

### Statistical methods

Relative cell fractions (proportions) of CD4 T, CD8 T, B-cells, NK cells, monocytes, and neutrophils, were estimated via our validated deconvolution algorithm [13]. These immune cell fractions and the absolute counts were analyzed as continuous variables. Absolute counts for specific immune cell subsets also were grouped for analyses based on clinically meaningful thresholds. Measures of CD4/CD8, NLR and LMR, previously associated with disease prognosis [3, 16, 17], were also examined. Analyses were conducted within glioma patients and for glioma patients compared with control participants (cell fractions only). Associations with demographic, clinical and tumor characteristics were examined using non-parametric Kruskal–Wallis methods for continuous variables and chi-square statistics for categorical variables. Follow-up pairwise analyses assessed differences within groups when overall analyses showed statistically significant effects. Box and whisker plots were used to graphically display the association between immune cell factors and clinical/tumor factors. Analyses stratified by age at enrollment (< 58 and ≥ 58 years based on our prior work showing an interaction of patient age with immune profiles in glioma survival [4]) and dexamethasone use within 24 h before blood draw (hereafter referred to as DEX use), and within specific diagnostic groups and WHO 2016 classifications, were limited by small numbers of cases. Results were considered statistically significant for two-sided p-values < 0.05.

## Results

This study uses 139 of 243 patients who were eligible and contacted for enrollment in the IPS, for whom pre-surgery-blood samples were obtained. Blood samples were collected a median of 1 day before surgery (range 0–71 days).

### Presurgery immune cell subset profiles and patient characteristics

Glioma patients were largely white (89.9%) and male (61.2%) with a median age of 51 years (Table 1). There were 44 patients with IDHmt 1p19q co-deleted oligodendroglioma, 20 with IDHmt astrocytoma, 15 with IDHmt GBMs, and 60 with IDHwt GBM (Supplemental Table 2). Among the 15 IDHmt GBM, 7 were patients with initial LrGG that recurred as GBM. Nearly half of all glioma patients reported DEX use.

**Table 1:** Participant characteristics by case–control status for the 139 Immune Profile Study (IPS) glioma patients and 454 Adult Glioma Study (AGS) controls

Characteristic	AGS control (N = 454)	IPS glioma patient (N = 139)	Total (N = 593)	p value
Gender				0.161^a^
Female	207 (45.6%)	54 (38.8%)	261 (44.0%)	
Male	247 (54.4%)	85 (61.2%)	332 (56.0%)	
White				< 0.001^a^
Non-white	132 (29.1%)	14 (10.1%)	146 (24.6%)	
White	322 (70.9%)	125 (89.9%)	447 (75.4%)	
Age 1st diagnosis				
N	0	57	57	
Mean (SD)	NA	39.00 (13.70)	39.00 (13.70)	
Median	NA	36.00	36.00	
IQR	NA	17.00	17.00	
Q1, Q3	NA	29.00, 46.00	29.00, 46.00	
Age at enrollment				0.440^b^
N	454	139	593	
Mean (SD)	51.77 (15.53)	50.54 (15.40)	51.48 (15.49)	
Median	52.00	51.00	52.00	
IQR	22.00	24.00	24.00	
Q1, Q3	41.00, 63.00	38.00, 62.00	39.00, 63.00	
Dexamethasone at draw				< 0.001^a^
N-Miss	6	0	6	
No	447 (99.8%)	76 (54.7%)	523 (89.1%)	
Yes	1 (0.2%)	63 (45.3%)	64 (10.9%)	
Tumor grade				
N-Miss	454	0	454	
2	0	35 (25.2%)	35 (25.2%)	
3	0	27 (19.4%)	27 (19.4%)	
4	0	77 (55.4%)	77 (55.4%)	
WHO 2016 Classification				
N-Miss	454	0	454	
IDH mutant (mt) 1p19q codel oligodendroglioma	0	44 (31.7%)	44 (31.7%)	
IDHmt Astrocytoma	0	20 (14.4%)	20 (14.4%)	
IDHmt Glioblastoma (GBM)	0	15 (10.8%)	15 (10.8%)	
IDH wild-type GBM	0	60 (43.2%)	60 (43.2%)	
Diagnosis group				
New GBM	0 (0.0%)	67 (48.2%)	67 (11.3%)	
New lower grade glioma (LrGG)	0 (0.0%)	29 (20.9%)	29 (4.9%)	
Recurrent LrGG- > LrGG	0 (0.0%)	33 (23.7%)	33 (5.6%)	
Recurrent LrGG- > GBM	0 (0.0%)	10 (7.2%)	10 (1.7%)	
Control	454 (100.0%)	0 (0.0%)	454 (76.6%)	

104 Eligible glioma patients were not included: 73 declined to participate, 22 enrolled but did not provide a pre-surgery blood sample, 5 had missing information or QC testing and 4 had samples that were not arrayed at the time of analysis

^a^Pearson’s Chi-squared test

^b^Kruskal–Wallis rank sum test

Comparisons of relative cell fractions between controls and glioma patients showed statistically significant lower cell fractions for glioma patients for all immune cell subsets except for neutrophils which were statistically significantly higher (all p-values < 0.001, Table 2).

**Table 2:** Descriptive statistics for immune cell subset fractions and absolute counts estimated in pre-surgery blood samples collected from 139 Immune Profile Study (IPS) glioma patients and from blood collected at time of interview for 454 Adult Glioma Study (AGS) controls

Immune cell subset	AGS control (N = 454)	IPS glioma patient (N = 139)	p value
Natural Killer cell %			< 0.001^b^
N	454	139	
Mean (SD)	0.05 (0.03)	0.04 (0.03)	
Median	0.05	0.04	
IQR	0.03	0.03	
Q1, Q3	0.03, 0.06	0.02, 0.06	
CD4 cell %			< 0.001^b^
N	454	139	
Mean (SD)	0.16 (0.06)	0.10 (0.07)	
Median	0.16	0.10	
IQR	0.08	0.12	
Q1, Q3	0.12, 0.20	0.04, 0.15	
CD8 cell %			< 0.001^b^
N	454	139	
Mean (SD)	0.10 (0.06)	0.07 (0.04)	
Median	0.09	0.06	
IQR	0.07	0.07	
Q1, Q3	0.06, 0.13	0.03, 0.09	
B cell %			< 0.001^b^
N	454	139	
Mean (SD)	0.06 (0.03)	0.04 (0.02)	
Median	0.05	0.04	
IQR	0.03	0.03	
Q1, Q3	0.04, 0.07	0.02, 0.05	
Monocyte %			< 0.001^b^
N	454	139	
Mean (SD)	0.08 (0.03)	0.06 (0.03)	
Median	0.07	0.06	
IQR	0.03	0.04	
Q1, Q3	0.06, 0.09	0.05, 0.08	
Neutrophil %			< 0.001^b^
N	454	139	
Mean (SD)	0.58 (0.12)	0.70 (0.15)	
Median	0.58	0.67	
IQR	0.14	0.25	
Q1, Q3	0.51, 0.66	0.58, 0.83	
Total cell count			
N	0	139	
Mean (SD)	NA	8781.43 (4403.07)	
Median	NA	7900.00	
IQR	NA	5703.67	
Q1, Q3	NA	5581.33, 11,285.00	
Total cells grouped			
< 10 K	0	96 (69.1%)	
≥ 10 K	0	43 (30.9%)	
Absolute CD4 count			
N	0	139	
Mean (SD)	NA	730.484 (480.77)	
Median	NA	671.90	
IQR	NA	659.10	
Q1, Q3	NA	347.43, 1006.52	
CD4 grouped			
< 500	0	51 (36.7%)	
≥ 500	0	88 (63.3%)	
CD4 grouped			
< 200	0	19 (13.7%)	
≥ 200	0	120 (86.3%)	
Absolute CD8 count			
N	0	139	
Mean (SD)	NA	478.80 (301.01)	
Median	NA	460.20	
IQR	NA	397.73	
Q1, Q3	NA	249.41, 647.14	
Absolute B cell count			
N	0	139	
Mean (SD)	NA	329.03 (213.25)	
Median	NA	279.85	
IQR	NA	183.33	
Q1, Q3	NA	196.94, 380.27	
Absolute Natural Killer cell count			
N	0	139	
Mean (SD)	NA	322.50 (202.74)	
Median	NA	281.56	
IQR	NA	186.36	
Q1, Q3	NA	199.56, 385.92	
Absolute lymphocyte count			
N	0	139	
Mean (SD)	NA	1860.82 (925.39)	
Median	NA	1718.86	
IQR	NA	1293.56	
Q1, Q3	NA	1119.20, 2412.76	
Absolute lymphocytes grouped			
< 1 K	0	28 (20.1%)	
≥ 1 K	0	111 (79.9%)	
Absolute neutrophil count			
N	0	139	
Mean (SD)	NA	6532.17 (4313.68)	
Median	NA	5095.83	
IQR	NA	5379.93	
Q1, Q3	NA	3256.90, 8636.83	
Absolute neutrophils grouped			
< 8870	0	106 (76.3%)	
≥ 8870	0	33 (23.7%)	
Absolute monocyte			
N	0	139	
Mean (SD)	NA	505.32 (272.30)	
Median	NA	445.23	
IQR	NA	283.13	
Q1, Q3	NA	337.14, 620.26	
CD4/CD8 ratio			< 0.001^b^
N	454	139	
Mean (SD)	2.19 (1.84)	1.74 (1.45)	
Median	1.75	1.47	
IQR	1.36	1.02	
Q1, Q3	1.21, 2.57	1.01, 2.03	
Neutrophil lymphocyte ratio			< 0.001^b^
N	454	139	
Mean (SD)	1.84 (1.14)	4.97 (5.20)	
Median	1.57	2.69	
IQR	1.05	4.49	
Q1, Q3	1.16, 2.21	1.65, 6.14	
Neutrophil lymphocyte ratio grouped			< 0.001^a^
< 4	438 (96.5%)	91 (65.5%)	
≥ 4	16 (3.5%)	48 (34.5%)	
Lymphocyte monocyte ratio			< 0.001^b^
N	454	139	
Mean (SD)	5.19 (2.16)	4.17 (1.90)	
Median	4.79	4.22	
IQR	2.61	2.96	
Q1, Q3	3.75, 6.36	2.64, 5.61	
Total lymphocyte %			< 0.001^b^
N	454	139	
Mean (SD)	0.37 (0.11)	0.25 (0.13)	
Median	0.37	0.25	
IQR	0.14	0.22	
Q1, Q3	0.29, 0.44	0.14, 0.35	

Absolute counts not determined for AGS controls therefore no statistical comparisons for immune cell measures based on absolute counts

^a^Pearson’s Chi-squared test

^b^Kruskal–Wallis rank sum test

### Pre-surgery immune profiles differ by glioma WHO 2016 Classification and control status

Patients with IDHwt GBM were older (median age at first diagnosis and age at enrollment were 59.5 years and 64.5 years respectively) than other glioma participants (median age 27 to 36 years and 35 to 46 years, respectively; Supplemental Table 1) and were more likely to report DEX use.

**Fig. 1:**
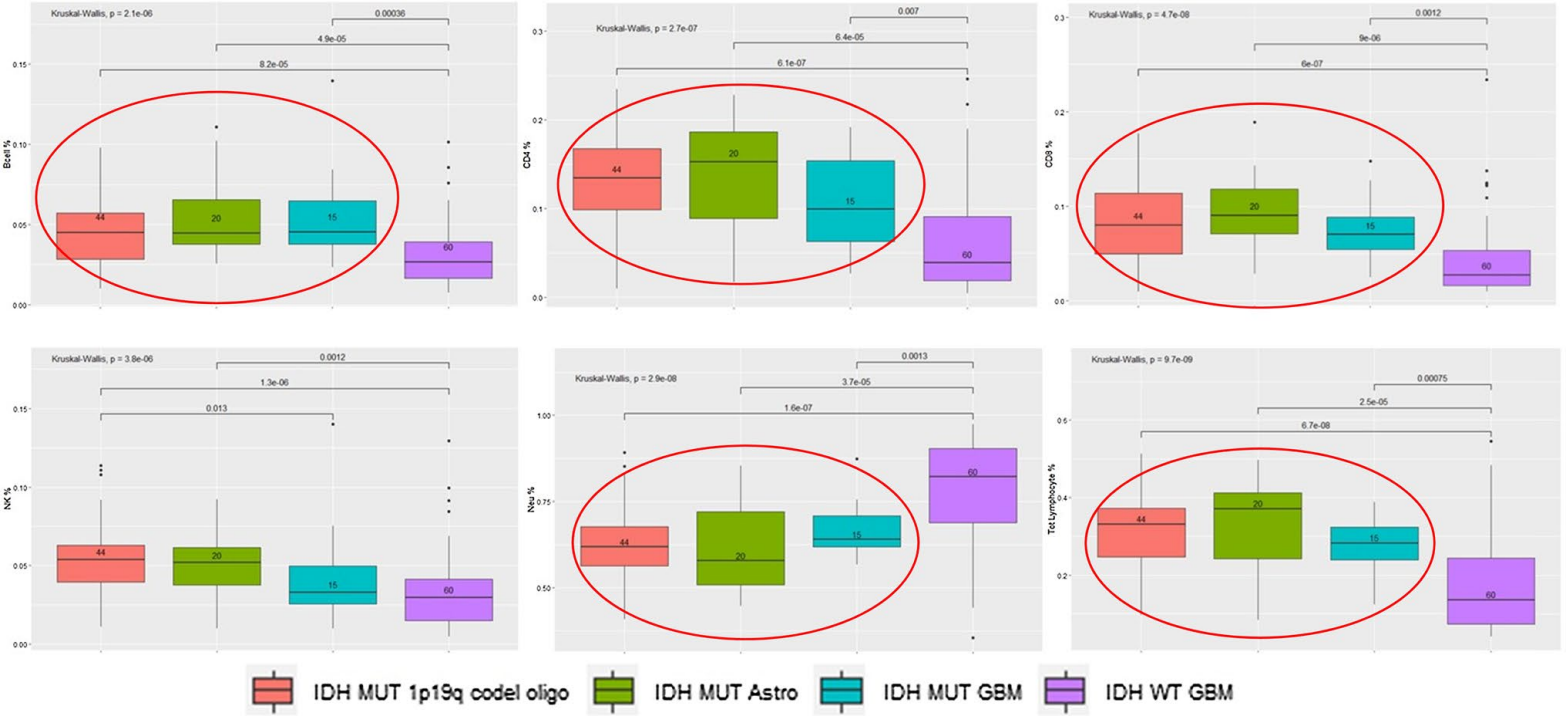
Distribution of specific cell subtype fractions by glioma WHO 2016 Classification among Immune Profile Study (IPS) Glioma patients. Boxplots of immune cell subset fractions [B cell, CD4 T cell (CD4), CD8 T cell (CD8), NK cells, Neutrophils, Total Lymphocytes] for Immune Profile Study (IPS) glioma patients grouped by WHO 2016 Glioma Classification. The median is represented by the solid line within each box, each box represents 50% of the data for that group, the vertical line emerging above the box represents the top 25% of the data spread and the vertical line emerging from the bottom of each box represents the lowest 25% of the data spread. The number of patients in each group are provided in the boxplot. The Kruskal–Wallis p-value is for the test of an overall difference in immune cell subset fraction among the four groups. p-Values for pairwise comparisons are depicted by brackets with the groups at each end of the bracket being compared. In general, immune cell fractions differed between IDHwt GBM and other glioma subtypes (lower in IDHwt GBM apart from neutrophils which were higher) whereas differences were not observed between other glioma subtypes (depicted by red circles in cell subtype plot)

**Fig. 2:**
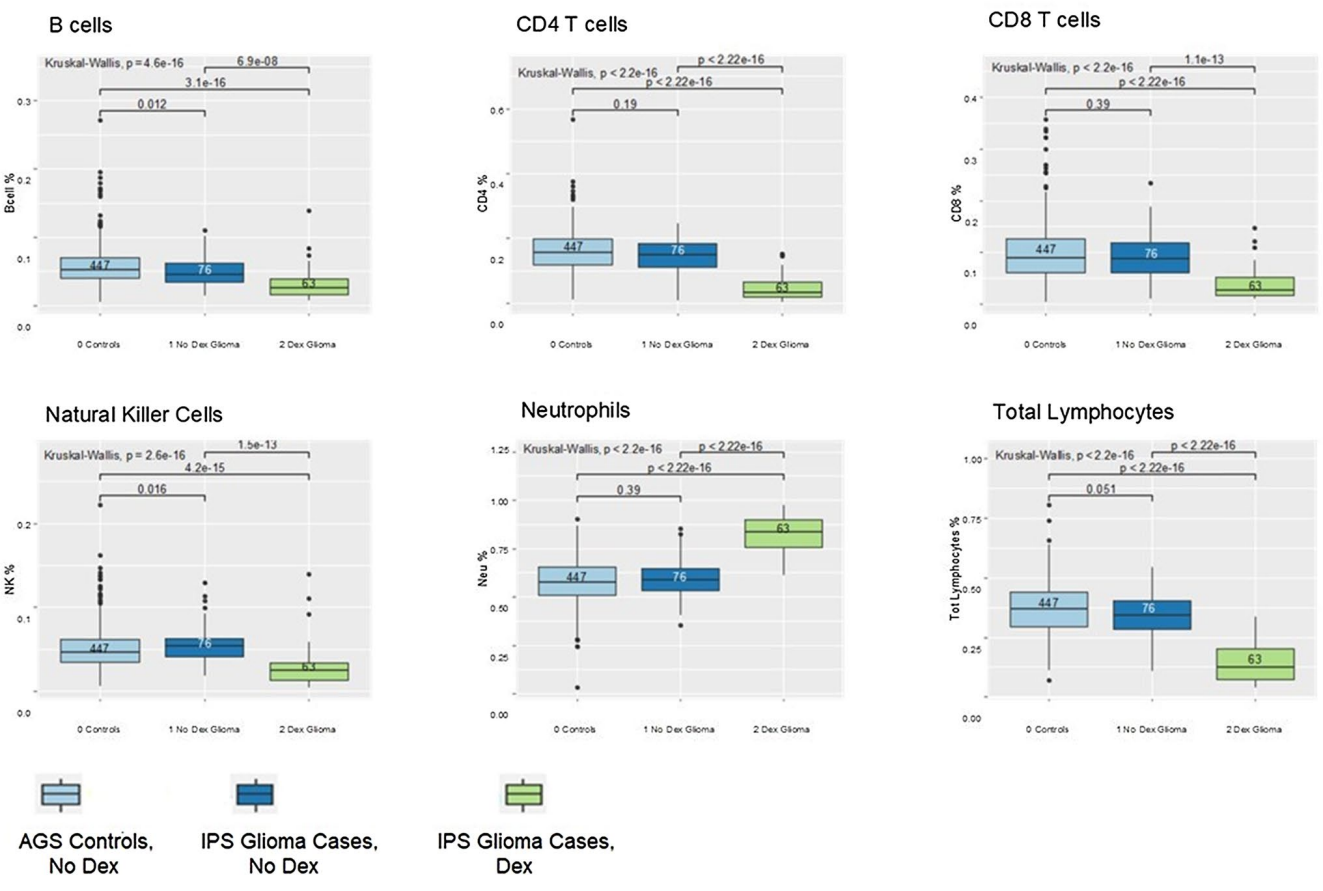
Boxplots for the comparisons of immune cell subset fractions among Immune Profile Study (IPS) glioma patients and Adult Glioma Study (AGS) controls by dexamethasone use at the time of blood draw. Boxplots of the distribution of fractions of specific immune cell subsets (B cells, CD4 T cells, CD8 T cells, NK cells, Neutrophils, Total Lymphocytes) for IPS glioma cases and AGS controls by dexamethasone use. The median is represented by the solid line within each box, each box represents 50% of the data for that group, the line emerging above the box represents the top 25% of the data spread and the line emerging from the bottom of each box represents the lowest 25% of the data spread. The number of persons in each group are provided in the boxplot. The Kruskal–Wallis p-value is for the test of an overall difference among the three groups (AGS controls, IPS glioma cases who used dexamethasone, IPS glioma cases who did not use dexamethasone) within each immune cell subset. Pairwise comparisons are depicted by brackets with the groups at each end of the bracket being compared and the p-value for that difference indicated on top of the bracket

Overall analyses of cell subtypes indicated that the fractions and absolute counts of all specific cell subsets differed by WHO 2016 classification with the exception of absolute NK cell and absolute monocyte levels (Supplemental Table 2). Follow-up pairwise comparisons of cell fractions (Supplemental Table 3) showed that except for higher neutrophil fractions, all other cell fractions tended to be lower in those with IDHwt GBMs than in those with IDHmt 1p19q co-deleted oligodendroglioma and IDHmt astrocytoma (Fig. 1). In analyses that included controls, results were consistent with those among glioma patients only (Supplemental Tables 2, 3) and notable for the higher cell fraction levels in controls compared with glioma subtypes with the exception of lower neutrophil levels (Fig. 2).

Results from follow-up pairwise comparisons of absolute cell counts were consistent with differences observed for cell fraction analyses (Supplemental Table 3) with the notable exception of higher absolute B-cell counts in IDHmt GBM. In addition, analyses of grouped absolute CD4 T cell, absolute lymphocyte, absolute neutrophil and total cell counts, and of NLR and LMR, each showed an overall difference across WHO 2016 classification groups, largely driven by differences with IDHwt GBMs (data not shown).

### Pre-surgery blood immune cell subset profiles by glioma status are associated with DEX use

When glioma cases and controls were stratified by DEX use, overall results showed that cell subset fractions differed across the three groups (Supplemental Table 4). Glioma patients who used DEX had the lowest CD4 T, CD8 T, B, NK, monocyte and total lymphocyte cell fractions as well as CD4/CD8 ratio and LMR, and the highest neutrophil fractions and NLR (Fig. 2; Supplemental Table 5). Interestingly, cell subset fractions did not differ between control participants and glioma patients who did not use DEX (Fig. 2). Compared to those who did not use DEX, glioma patients who used DEX were also more likely to have a CD4 count < 200, CD4 count < 500, ANC ≥ 8870, ALC < 1000, total cell count ≥ 10,000 and an NLR ≥ 4 (all p < 0.001, Supplemental Table 4).

### Pre-surgery immune cell subset profiles vary by glioma WHO 2016 Classification and DEX use

**Fig. 3:**
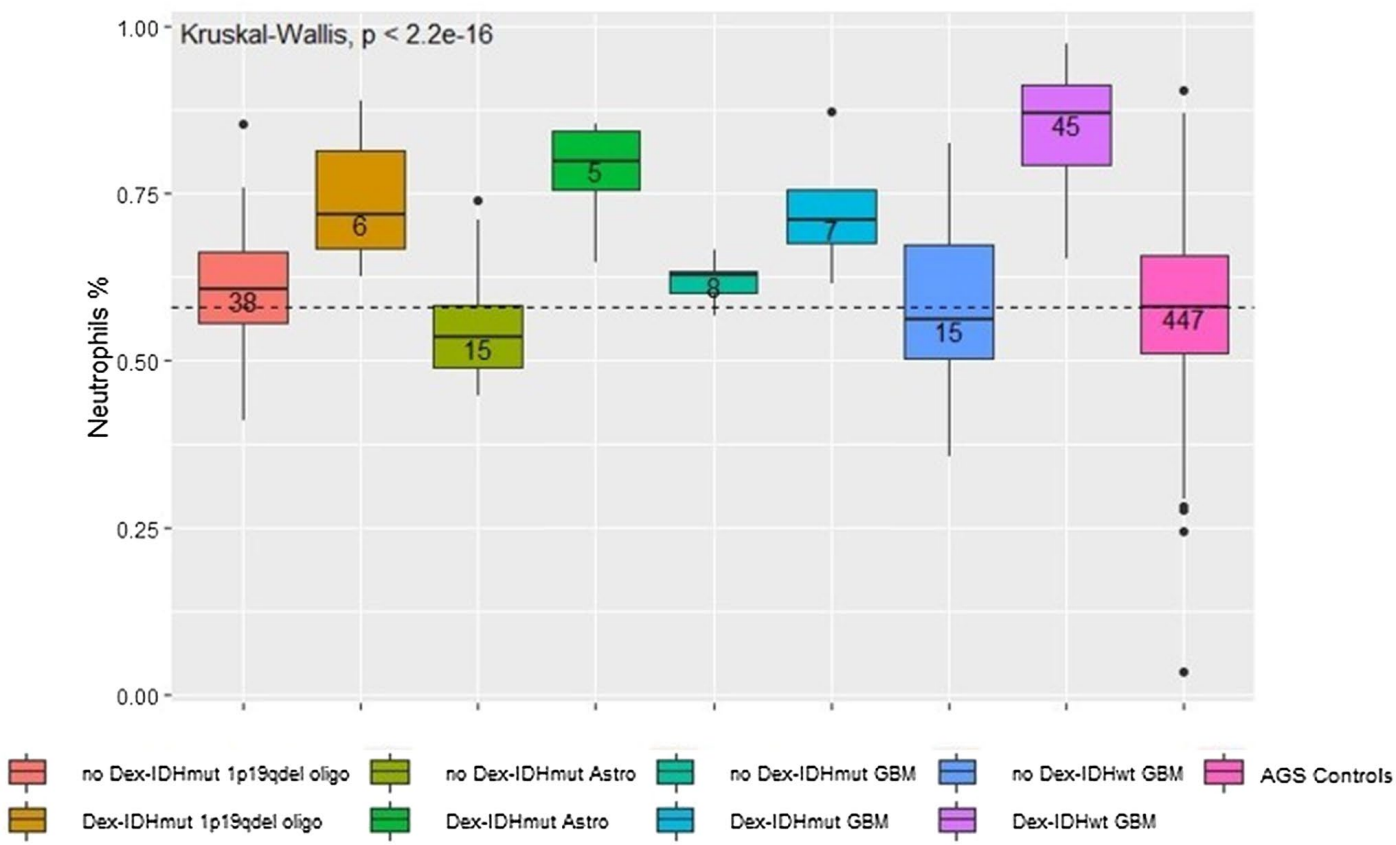
Neutrophil cell fractions for dexamethasone use at blood draw by WHO 2016 Classification of Immune Profile Study (IPS) Glioma Cases, and Adult Glioma Study (AGS) Controls (no DEX). Boxplots of neutrophil fractions for IPS glioma cases grouped by WHO 2016 Glioma Classification and DEX status, and AGS controls who did not use DEX. The median is represented by the solid line within each box, each box represents 50% of the data for that group, the line emerging above the box represents the top 25% of the data spread and the line emerging from the bottom of each box represents the lowest 25% of the data spread. The horizontal broken line represents the median neutrophil fraction among AGS controls. The number of patients or controls in each group are provided in the boxplot. The Kruskal–Wallis p-value is for the test of an overall difference in neutrophil fraction among the nine groups

**Fig. 4:**
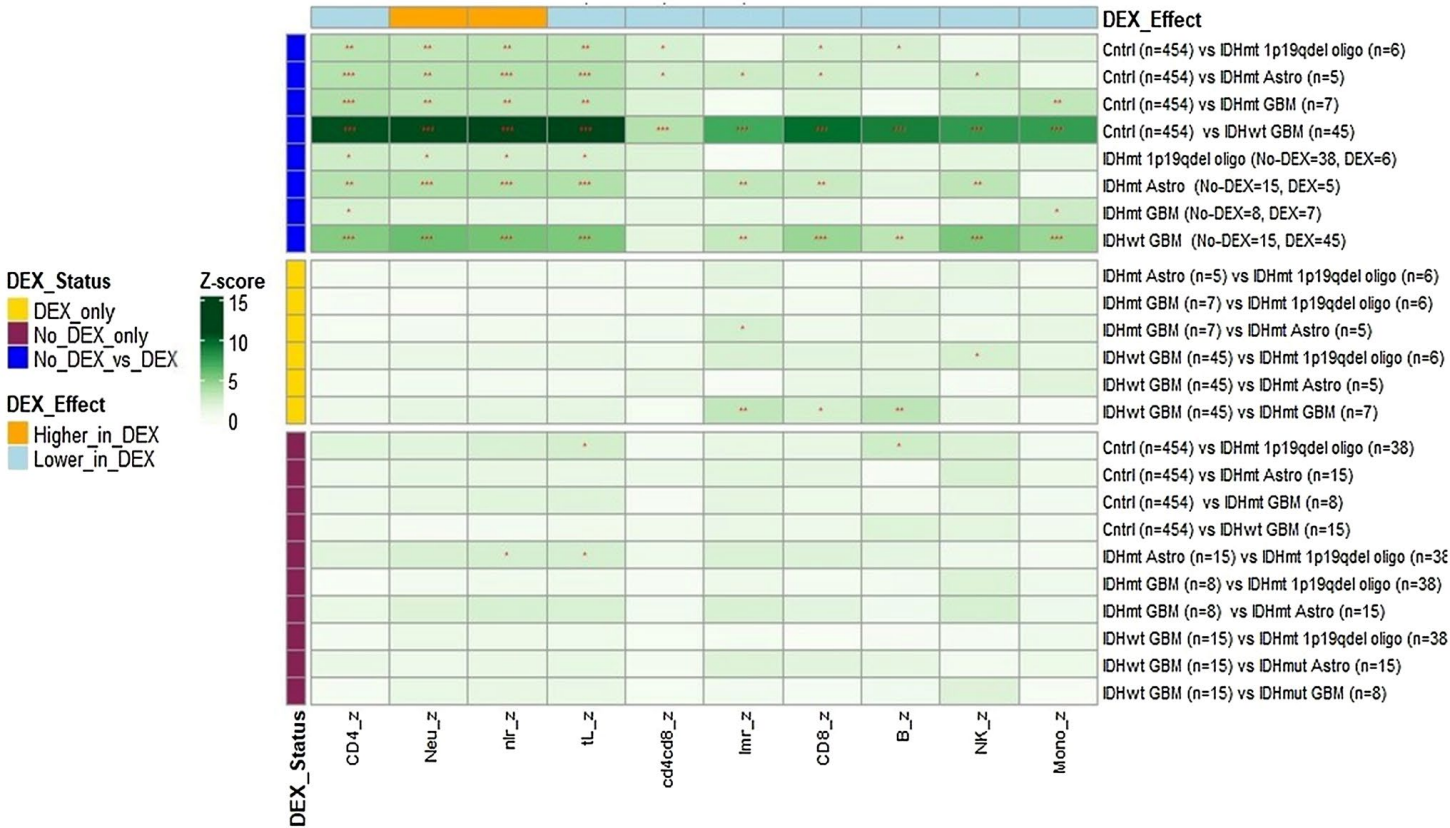
Heatmap of Z-scores from pairwise comparisons of immune cell subset fractions among Adult Glioma Study (AGS) controls (none taking DEX) and Immune Profiles Study (IPS) Glioma patients by WHO 2016 Classification and DEX use at blood draw. Heatmap of Z-score values from pairwise comparisons (Dunn test) of immune cell subset fractions among IPS Glioma patients classified per WHO 2016 Classification and DEX status, and AGS controls (no DEX). Row labels (right side of plot) indicate the groups being compared (controls and glioma cases) and the number in each group. Column labels (bottom of plot) indicate the specific immune cell subset Z-score that is being depicted for the comparisons made (CD4_z = CD4 T cells, Neu_z = Neutrophils, nlr_z = Neutrophil to Lymphocyte Ratio, tL_z = Total Lymphocytes, lmr_z = Lymphocyte to Monocyte Ratio, CD8_z = CD8 T cells, B_z = B cells, NK_z = Natural Killer cells, Mono_z = Monocytes). Each block represents the Z score for the comparison of an immune cell subset fraction between two groups of patients noted in the right-hand margin of the map e.g., the left-most square in the top row represents the Z score for the comparison of CD4 T cell fraction values between AGS controls (no DEX) and IPS glioma patients with IDHmt 1p19q co-deleted oligodendroglioma (used DEX). Darker green blocks represent higher Z scores and as shown, most statistically significant differences (denoted as *p < 0.05, **p < 0.01, ***p < 0.001) were observed for comparisons of no DEX versus DEX groups (top eight rows). Effects were greatest and similar for immune cell subsets CD4 T cell, Neutrophil, Neutrophil to Lymphocyte ratio (NLR), and total lymphocytes (tL), and for AGS controls compared with IPS glioma patients with IDHwt GBM who used DEX. Overall, immune cell subset fraction values were lower among DEX users regardless of immune cell subtype with the exception of neutrophil fractions and NLR which were higher among DEX users as denoted by the color coding in the top row (light blue indicates that, when significant, values were lower in DEX users than in non-DEX users whereas, orange indicates that values were higher in DEX users than in non-DEX users). Among DEX users (middle 6 rows), cell fractions in IPS patients with IDHwt GBM were lower when statistically significant differences were observed. Among those who did not use DEX (bottom 10 rows), cell fractions in AGS controls were higher, and IDHmt astrocytoma NLR was higher and Total Lymphocytes lower when statistically significant

Cell subset fractions and absolute counts were assessed for the four WHO 2016 Classification groups and DEX use (Supplemental Table 6). There were differences across these eight groups for all cell subsets apart from absolute B, NK and monocyte cell counts. Follow-up pairwise analyses showed a distinct separation and significant differences in immune cell subset fractions between patient groups (Figs. 3, 4); mainly between those who were and were not using DEX, e.g., particularly controls (no DEX) and glioma patients using DEX. Notable were comparisons with IDHwt GBM (all fractions except for CD4/CD8 ratio) and with IDHmt astrocytoma (all fractions except B cells and monocytes, and CD4/CD8 ratio). Difference patterns were similar, with lower CD4 T and total lymphocyte cell fractions, and higher neutrophil fractions and NLR in DEX users (Fig. 4). Few differences were observed in immune cell subset fractions when comparisons were restricted to patients and controls who did not use DEX, or glioma patients who used DEX; in DEX users, CD8 T and B cell fractions, and LMR were lower in IDHwt compared to IDHmt GBM patients (Fig. 4).

Results from pairwise comparisons of absolute counts were similar to those for cell fractions with most differences observed between those who were and were not using DEX (data not shown). Again, IDHwt GBM patients using DEX tended to have lower cell counts (especially compared to patients with IDHmt 1p19q co-deleted oligodendroglioma not using DEX), apart from higher neutrophil counts, compared to other glioma subtype patients who were not using DEX. Additionally, a greater proportion of IDHwt GBM patients who used DEX had the following: (1) ANC count ≥ 8870, total cell count ≥ 10,000, and CD4 count < 500 compared to: IDHmt 1p19q co-deleted oligodendroglioma, IDHmt GBM and IDHwt GBM who did not use DEX; (2) CD4 count < 200 compared to: IDHmt 1p19q co-deleted oligodendroglioma who did not use DEX; (3) ALC count < 1000 compared to: IDHmt astrocytoma who used DEX, and IDHmt 1p19q co-deleted oligodendroglioma, IDHmt and IDHwt GBM who did not use DEX; and (4) NLR ≥ 4: compared to: IDHmt 1p19q co-deleted oligodendroglioma regardless of DEX use, IDHmt GBM who used DEX, and IDHwt GBM who did not use DEX.

### Description of IDHmt GBMs that were recurrences of LrGG

Nearly 50% of patients with IDHmt GBMs were recurrent LrGG (7/15). Comparing new to recurrent IDHmt GBM showed no differences in demographic or tumor characteristics. However, new IDHmt GBM patients had lower monocyte and total lymphocyte fractions (p = 0.049, 0.037 respectively), and higher neutrophil fractions and NLR (p = 0.028, 0.037; respectively) than patients with recurrent IDHmt GBM. Exploration of DEX use (29% of recurrent, 62% of new IDHmt GBM patients) was limited by small group size (< 5 cases/group) but suggested an association with DEX. New patients who used DEX had (1) higher NLR and lower total lymphocyte fractions than new patients who did not use DEX (p = 0.02 for both) and recurrent patients regardless of DEX use (no DEX p = 0.009, DEX p = 0.03; for both); (2) higher neutrophil fractions than new or recurrent patients who did not use DEX (p = 0.03, 0.004; respectively) and; (3) lower monocyte fractions than recurrent patients who did not use DEX (p = 0.005). No other differences in cell fractions or absolute counts were observed.

### Detailed characteristics of DEX use by WHO 2016 Classification

Patients with IDHwt GBM were more likely than patients with other glioma subtypes to report using DEX (75% vs: 48% for IDHmt GBM, 25% for IDHmt astrocytoma, 14% for IDHmt 1p19q co-deleted oligodendroglioma; Supplemental Table 7). In analyses restricted to DEX users, glioma subtype was associated with number of doses/day (p = 0.004, highest among IDHwt GBM) and somewhat associated with average-dose/day (p = 0.08, highest among IDHmt GBM), but not with total number of days used or mg/dose.

Analyses among IDHwt GBM patients showed that DEX use was associated with lower cell counts and fractions except for CD4/CD8 ratio, absolute B and monocyte cell counts (data not shown).

### Relationship between older age and pre-surgery immune cell subsets when stratified by GBM vs. non-GBM and DEX use

In analyses stratified by DEX use and LrGG vs. GBM, older GBM patients were statistically significantly more likely to have lower absolute CD4 T cell (< 500 or < 200 cells/μl) and lymphocyte counts, and increased neutrophils and NLR compared with younger patients (Supplemental Table 8). However, few cases in other substrata precluded meaningful analyses and interpretation.

## Discussion

The impacts of glioma on systemic cellular immune parameters are complex and include the potential interplay of tumor secreted and host inflammatory factors, immunosuppressive corticosteroid and chemoradiation therapies. A yet unanswered question is whether there are prognostically important features of the peripheral immune profile prior to therapeutic interventions. Studying patients prior to surgery and chemotherapy, as done here, reduces several potent sources of variation capable of modifying systemic immunity. Considering this, we sought to ask whether blood immune profiles are altered at baseline and to what extent alterations may be attributed to dexamethasone exposure or tumor subtype prior to surgical resection. Here we estimated leukocyte subtypes using a validated methylation deconvolution method that has an improved accuracy over previous deconvolution methods [4, 13, 15, 18], has been applied in other studies [4, 19] and whose approach has compared favorably to other methods [20].. We show for the first time that pre-surgery immune subset profiles vary by glioma subtype; most notably, compared with other glioma subtypes, patients with IDHwt GBM had lower levels of all immune subsets except neutrophils, which were higher. We show that immune subsets were higher, except neutrophils which were lower, in controls than in glioma patients. Dexamethasone use at the time of blood draw is strongly associated with frequencies and proportions of many immune cell types.

Across all glioma subtypes, dexamethasone exposure was associated with significant alterations in cell proportions and concentrations. GBM patients had the highest frequency and average dose of dexamethasone use and the most divergent immune subtypes. Further analyses in larger studies are needed to confirm findings especially for the less common glioma types. If possible, immune measures taken before dexamethasone use begins could be very helpful to disentangle the effects of dexamethasone from tumor related immune suppression and to clarify the complex association between tumor immune infiltrates, such as monocyte-derived M2 tumor-associated macrophages that promote GBM tumor growth [21], and blood immune cell profiles.

Dexamethasone use has been associated with significant lymphopenia [22, 23] in patients with GBM, especially for CD4 T cells [22]. In addition, neutrophilia and high NLR have been reported among glioma patients compared to controls with the highest levels among glioma patients with higher grade tumors. NLR > 4 has been related to poorer prognosis [23–27], and steroid treatment has been reported to further increase neutrophilia during pretreatment and correlated with poorer survival [28]. There are few published data related to other cell subsets but several do not support an association of glioma subtypes, glioma risk or prognosis with NK cells [22, 29], CD8 T cells [29], monocytes [22, 23], and B cells [22, 29]. However, there are some reports of lower CD8 T cells in GBM patients regardless of dexamethasone use [22], higher monocyte levels in high grade vs low grade glioma, and in glioma vs. controls [26, 30]. In contrast, there are reports of lower monocyte levels in overall glioma and in GBMs compared to controls [31].

Finally, we explored pre-surgery blood immune cell abnormalities among older compared with younger dexamethasone treated patients based on finding an interaction on survival with age and post-diagnosis immune subtypes [4]. Both cumulative and average daily doses of dexamethasone were similar in the two age strata (< vs. > 58). Despite these similar exposures, there were consistently more abnormalities in T cell, total lymphocyte and neutrophil parameters among older patients taking dexamethasone. When additionally stratified by lower grade glioma vs. GBM, these relationships only held among GBM patients. However, because LrGG patients are younger at diagnosis and less likely to have been taking dexamethasone, we were unable to adequately assess the effects within LrGG. These results, which are not confounded by chemoradiation or extent of surgery, suggest age-related changes in patient susceptibility to the immune modulating effects of dexamethasone. Age is a prominent factor influencing both glioma risk and survival. Furthermore, age related changes in immune response, termed immunosenescent, are well documented [32–37]. The desirability of limiting corticosteroid exposure in glioma patient management has been voiced elsewhere [38]; the current results point to an additional concern for older patients receiving dexamethasone, who may experience more pronounced changes in circulating immune cells. To better understand how or whether this age relationship with dexamethasone use varies by glioma subtype, studies with larger numbers of LrGG participants are needed.

## Conclusion

Compared to controls, glioma patients had statistically significant lower cell fractions of immune cell subsets except for neutrophils which were significantly higher. Patients who used dexamethasone were more likely to have abnormal immune cell subset levels based on clinical thresholds, particularly for CD4 T cell, neutrophil and lymphocyte counts which exploratory analyses suggest were driven by effects among older GBM patients. However, age and dexamethasone use confound the association between immune cell subset profiles and glioma subtypes. Therefore, further analyses in longitudinal populations with a larger number of LrGG patients are warranted to determine whether this preponderance of associations within the IDHwt GBM group in our analyses is due to sample size or true differences in immune profiles across glioma subtypes.

## Supplementary Information

Below is the link to the electronic supplementary material.

Supplementary file1 (DOCX 120 kb)

## Declarations

### Prior Presentations

This research was presented as an on-demand pre-recorded oral presentation at the Society for Neuro-Oncology (SNO) Annual Meeting (November 19–22, 2020) and the Brain Tumor Epidemiology Consortium Annual Meeting (June 22–25, 2021).
