# Supplementary material for: Pre-surgery immune profiles of adult glioma patients
Source: J Neurooncol. 2022 Jun 18;159(1):103–15. doi: 10.1007/s11060-022-04047-y (PMC9325836; doi:10.1007/s11060-022-04047-y)
Supplement: Supplementary file 1 — Supplementary file1 (DOCX 120 kb) [file 11060_2022_4047_MOESM1_ESM.docx]

**Pre-surgery Immune Profiles of Adult Glioma Patients,**

Paige M Bracci^1^, Terri Rice^2^, Helen M Hansen^2^, Stephen S Francis^2^, Sean Lee^2^, Lucie S McCoy^2^, Pavan P Shrestha^2^, Gayathri Warrier^2^, Jennifer L Clarke^2,3^, Annette M Molinaro^1,2^, Jennie W Taylor^2,3^, John K Wiencke^2^, Margaret R Wrensch^2^

Author Affiliations

^1^Department of Epidemiology and Biostatistics, UCSF, San Francisco CA.

^2^Department of Neurological Surgery, UCSF, San Francisco C

^3^Department of Neurology, UCSF, San Francisco CA.

**Corresponding Author:**

Paige Bracci, PhD, MPH, MS

1450 3rd Street, San Francisco, CA 94158

Phone: (415) 476-2300, Email: [paige.bracci@ucsf.edu](mailto:paige.bracci@ucsf.edu)

**Supplement Table 1** Descriptive Statistics for Immune Cell Subset fractions (proportions) and absolute counts estimated in pre-surgery bloods collected from by WHO 2016 classification for 139 Immune Profile Study (IPS) glioma participants and from blood collected at time of interview for 454 Adult Glioma Study (AGS) controls

|  | 1 IDHmt 1p19qdel oligo (N=44) | 2 IDHmt Astro (N=20) | 3 IDHmt GBM (N=15) | 4 IDHwt GBM (N=60) | 5 AGS cntrl (N=454) | p value* |
| --- | --- | --- | --- | --- | --- | --- |
| **Gender** |  |  |  |  |  | 0.365/0.487 |
| F | 20 (45.5%) | 6 (30.0%) | 4 (26.7%) | 24 (40.0%) | 207 (45.6%) |  |
| M | 24 (54.5%) | 14 (70.0%) | 11 (73.3%) | 36 (60.0%) | 247 (54.4%) |  |
| **White** |  |  |  |  |  | < 0.001/0.367 |
| non-white | 7 (15.9%) | 1 (5.0%) | 2 (13.3%) | 4 (6.7%) | 132 (29.1%) |  |
| white | 37 (84.1%) | 19 (95.0%) | 13 (86.7%) | 56 (93.3%) | 322 (70.9%) |  |
| **Tumor grade** |  |  |  |  |  |  |
| 2 | 23 (52.3%) | 12 (60.0%) | 0 (0.0%) | 0 (0.0%) | 0 |  |
| 3 | 19 (43.2%) | 8 (40.0%) | 0 (0.0%) | 0 (0.0%) | 0 |  |
| 4 | 2 (4.5%) | 0 (0.0%) | 15 (100.0%) | 60 (100.0%) | 0 |  |
| **Diagnosis group** |  |  |  |  |  | < 0.001/<0.001 |
| 1 new gbm | 0 (0.0%) | 0 (0.0%) | 8 (53.3%) | 59 (98.3%) | 0 (0.0%) |  |
| 2 new lgg | 17 (38.6%) | 12 (60.0%) | 0 (0.0%) | 0 (0.0%) | 0 (0.0%) |  |
| 3 rec lgg, still lgg | 25 (56.8%) | 8 (40.0%) | 0 (0.0%) | 0 (0.0%) | 0 (0.0%) |  |
| 4 rec lgg, now gbm | 2 (4.5%) | 0 (0.0%) | 7 (46.7%) | 1 (1.7%) | 0 (0.0%) |  |
| 5 control | 0 (0.0%) | 0 (0.0%) | 0 (0.0%) | 0 (0.0%) | 454 (100.0%) |  |
| **Dexamethasone at draw** |  |  |  |  |  | < 0.001/<0.001 |
| N-Miss | 0 | 0 | 0 | 0 | 6 |  |
| No | 38 (86.4%) | 15 (75.0%) | 8 (53.3%) | 15 (25.0%) | 447 (99.8%) |  |
| Yes | 6 (13.6%) | 5 (25.0%) | 7 (46.7%) | 45 (75.0%) | 1 (0.2%) |  |
| **Dexamethasone in past mo.** |  |  |  |  |  | NA/<0.001 |
| No | 38 (86.4%) | 14 (70.0%) | 7 (46.7%) | 9 (15.0%) | 0 |  |
| Yes | 6 (13.6%) | 6 (30.0%) | 8 (53.3%) | 51 (85.0%) | 0 |  |
| **Age 1st Diagnosis** |  |  |  |  |  |  |
| N | 29 | 9 | 9 | 10 | 0 | NA/0.021 |
| Mean (SD) | 37.103 (8.432) | 31.778 (7.513) | 35.111 (14.287) | 54.500 (19.231) | NA |  |
| Median | 36.000 | 30.000 | 27.000 | 59.500 | NA |  |
| IQR | 9.000 | 9.000 | 24.000 | 32.250 | NA |  |
| Q1, Q3 | 34.000, 43.000 | 27.000, 36.000 | 22.000, 46.000 | 38.250, 70.500 | NA |  |
| **Age at enrollment** |  |  |  |  |  | < 0.001/<0.001 |
| N | 44 | 20 | 15 | 60 | 454 |  |
| Mean (SD) | 45.909 (9.894) | 37.100 (9.458) | 38.800 (13.056) | 61.350 (13.541) | 51.773 (15.527) |  |
| Median | 46.000 | 37.000 | 35.000 | 64.500 | 52.000 |  |
| IQR | 16.000 | 9.500 | 22.000 | 12.500 | 22.000 |  |
| Q1, Q3 | 38.000, 54.000 | 30.500, 40.000 | 28.000, 50.000 | 57.000, 69.500 | 41.000, 63.000 |  |

*Pearson Chi-sq for categorical variables, Kruskal-Wallis test for continuous factors; p value for comparisons including controls/p value for comparisons for cases only

**Supplemental Table 2** Descriptive Statistics for Immune Cell Subset fractions (proportions) and absolute counts estimated in pre-surgery bloods collected from for 139 Immune Profile Study (IPS) glioma participants stratified by WHO 2016 classification, and in blood collected at time of interview for 454 Adult Glioma Study (AGS) controls

|  | 1 IDHmt 1p19qdel oligo (N=44) | 2 IDHmt Astro (N=20) | 3 IDHmt GBM (N=15) | 4 IDHwt GBM (N=60) | 5 AGS cntrl (N=454) | p value* |
| --- | --- | --- | --- | --- | --- | --- |
| **Natural Killer cell %** |  |  |  |  |  | < 0.001/<0.001 |
| N | 44 | 20 | 15 | 60 | 454 |  |
| Mean (SD) | 0.055 (0.024) | 0.051 (0.023) | 0.042 (0.032) | 0.033 (0.024) | 0.051 (0.028) |  |
| Median | 0.054 | 0.052 | 0.033 | 0.030 | 0.047 |  |
| IQR | 0.023 | 0.024 | 0.024 | 0.026 | 0.028 |  |
| Q1, Q3 | 0.040, 0.063 | 0.038, 0.062 | 0.026, 0.050 | 0.015, 0.041 | 0.034, 0.062 |  |
| **CD4 T cell %** |  |  |  |  |  | < 0.001/<0.001 |
| N | 44 | 20 | 15 | 60 | 454 |  |
| Mean (SD) | 0.131 (0.056) | 0.138 (0.064) | 0.106 (0.057) | 0.066 (0.062) | 0.161 (0.064) |  |
| Median | 0.135 | 0.153 | 0.100 | 0.039 | 0.157 |  |
| IQR | 0.069 | 0.098 | 0.091 | 0.072 | 0.080 |  |
| Q1, Q3 | 0.099, 0.168 | 0.089, 0.187 | 0.063, 0.154 | 0.019, 0.091 | 0.118, 0.197 |  |
| **CD8 T cell %** |  |  |  |  |  | < 0.001/<0.001 |
| N | 44 | 20 | 15 | 60 | 454 |  |
| Mean (SD) | 0.081 (0.037) | 0.093 (0.039) | 0.074 (0.035) | 0.044 (0.043) | 0.100 (0.057) |  |
| Median | 0.080 | 0.090 | 0.070 | 0.027 | 0.089 |  |
| IQR | 0.064 | 0.047 | 0.034 | 0.037 | 0.066 |  |
| Q1, Q3 | 0.050, 0.114 | 0.071, 0.118 | 0.054, 0.089 | 0.017, 0.053 | 0.061, 0.126 |  |
| **B cell %** |  |  |  |  |  | < 0.001/<0.001 |
| N | 44 | 20 | 15 | 60 | 454 |  |
| Mean (SD) | 0.046 (0.020) | 0.053 (0.023) | 0.055 (0.029) | 0.031 (0.020) | 0.058 (0.029) |  |
| Median | 0.045 | 0.044 | 0.045 | 0.026 | 0.053 |  |
| IQR | 0.029 | 0.028 | 0.027 | 0.022 | 0.030 |  |
| Q1, Q3 | 0.028, 0.057 | 0.038, 0.065 | 0.038, 0.065 | 0.017, 0.039 | 0.040, 0.070 |  |
| **Monocyte %** |  |  |  |  |  | < 0.001/0.001 |
| N | 44 | 20 | 15 | 60 | 454 |  |
| Mean (SD) | 0.073 (0.025) | 0.071 (0.024) | 0.066 (0.025) | 0.054 (0.029) | 0.078 (0.027) |  |
| Median | 0.071 | 0.071 | 0.065 | 0.047 | 0.074 |  |
| IQR | 0.033 | 0.021 | 0.029 | 0.043 | 0.034 |  |
| Q1, Q3 | 0.056, 0.090 | 0.060, 0.080 | 0.050, 0.079 | 0.031, 0.074 | 0.060, 0.094 |  |
| **Neutrophil %** |  |  |  |  |  | < 0.001/<0.001 |
| N | 44 | 20 | 15 | 60 | 454 |  |
| Mean (SD) | 0.627 (0.101) | 0.609 (0.130) | 0.668 (0.080) | 0.785 (0.153) | 0.578 (0.116) |  |
| Median | 0.618 | 0.578 | 0.640 | 0.822 | 0.579 |  |
| IQR | 0.115 | 0.213 | 0.091 | 0.216 | 0.145 |  |
| Q1, Q3 | 0.563, 0.677 | 0.507, 0.720 | 0.618, 0.709 | 0.688, 0.904 | 0.510, 0.655 |  |
| **Total Cell Count** |  |  |  |  |  | NA/<0.001 |
| N | 44 | 20 | 15 | 60 | 0 |  |
| Mean (SD) | 6561.286 (2641.820) | 7527.832 (3647.669) | 8649.458 (3563.158) | 10860.390 (4934.935) | NA |  |
| Median | 6143.167 | 6277.133 | 7580.000 | 9616.667 | NA |  |
| IQR | 4197.250 | 3805.583 | 5113.833 | 6952.750 | NA |  |
| Q1, Q3 | 4367.750, 8565.000 | 5404.417, 9210.000 | 5961.000, 11074.833 | 7106.417, 14059.167 | NA |  |
| **Total Cells Grouped** |  |  |  |  |  | NA/<0.001 |
| N-Miss | 0 | 0 | 0 | 0 | 454 |  |
| <10K | 38 (86.4%) | 17 (85.0%) | 10 (66.7%) | 31 (51.7%) | 0 |  |
| >=10K | 6 (13.6%) | 3 (15.0%) | 5 (33.3%) | 29 (48.3%) | 0 |  |
| **Absolute CD4 count** |  |  |  |  |  | NA/<0.001 |
| N | 44 | 20 | 15 | 60 | 0 |  |
| Mean (SD) | 855.655 (504.674) | 859.586 (292.607) | 869.341 (585.516) | 560.943 (441.157) | NA |  |
| Median | 782.316 | 867.233 | 850.348 | 426.288 | NA |  |
| IQR | 624.584 | 416.302 | 583.601 | 543.784 | NA |  |
| Q1, Q3 | 537.057, 1161.640 | 646.424, 1062.726 | 399.453, 983.053 | 186.048, 729.832 | NA |  |
| **CD4 count group** |  |  |  |  |  | NA/<0.001 |
| N-Miss | 0 | 0 | 0 | 0 | 454 |  |
| <500 | 10 (22.7%) | 2 (10.0%) | 5 (33.3%) | 34 (56.7%) | 0 |  |
| >=500 | 34 (77.3%) | 18 (90.0%) | 10 (66.7%) | 26 (43.3%) | 0 |  |
| **CD4 count group** |  |  |  |  |  | NA/<0.001 |
| N-Miss | 0 | 0 | 0 | 0 | 454 |  |
| <200 | 3 (6.8%) | 0 (0.0%) | 0 (0.0%) | 16 (26.7%) | 0 |  |
| >=200 | 41 (93.2%) | 20 (100.0%) | 15 (100.0%) | 44 (73.3%) | 0 |  |
| **Absolute CD8 count** |  |  |  |  |  | NA/<0.001 |
| N | 44 | 20 | 15 | 60 | 0 |  |
| Mean (SD) | 484.789 (213.381) | 611.918 (228.530) | 676.729 (486.119) | 380.566 (282.916) | NA |  |
| Median | 474.452 | 583.179 | 513.624 | 274.495 | NA |  |
| IQR | 316.618 | 155.839 | 660.794 | 349.393 | NA |  |
| Q1, Q3 | 320.742, 637.359 | 505.784, 661.623 | 288.127, 948.922 | 177.630, 527.023 | NA |  |
| **Absolute B cell count** |  |  |  |  |  | NA/0.05 |
| N | 44 | 20 | 15 | 60 | 0 |  |
| Mean (SD) | 295.416 (197.097) | 368.887 (182.114) | 486.708 (341.334) | 300.979 (176.716) | NA |  |
| Median | 271.938 | 319.942 | 351.753 | 277.797 | NA |  |
| IQR | 163.299 | 166.427 | 391.608 | 205.309 | NA |  |
| Q1, Q3 | 170.962, 334.262 | 241.683, 408.110 | 257.027, 648.635 | 173.983, 379.293 | NA |  |
| **Absolute Natural Killer cell count** |  |  |  |  |  | NA/0.306 |
| N | 44 | 20 | 15 | 60 | 0 |  |
| Mean (SD) | 343.340 (183.525) | 347.295 (188.670) | 361.674 (386.683) | 289.154 (150.337) | NA |  |
| Median | 332.165 | 313.346 | 222.038 | 244.407 | NA |  |
| IQR | 194.416 | 230.328 | 126.579 | 158.572 | NA |  |
| Q1, Q3 | 212.028, 406.445 | 209.951, 440.279 | 193.314, 319.892 | 199.859, 358.431 | NA |  |
| **Absolute Lymphcyte count** |  |  |  |  |  | NA/0.001 |
| N | 44 | 20 | 15 | 60 | 0 |  |
| Mean (SD) | 1979.200 (856.377) | 2187.686 (694.738) | 2394.452 (1182.902) | 1531.641 (867.034) | NA |  |
| Median | 1886.773 | 2233.632 | 2206.359 | 1413.622 | NA |  |
| IQR | 1397.995 | 793.366 | 1199.778 | 1079.516 | NA |  |
| Q1, Q3 | 1217.238, 2615.232 | 1706.587, 2499.953 | 1687.604, 2887.382 | 838.219, 1917.735 | NA |  |
| **ALC group** |  |  |  |  |  |  |
| N-Miss | 0 | 0 | 0 | 0 | 454 |  |
| <1K | 6 (13.6%) | 0 (0.0%) | 1 (6.7%) | 21 (35.0%) | 0 |  |
| >=1K | 38 (86.4%) | 20 (100.0%) | 14 (93.3%) | 39 (65.0%) | 0 |  |
| **Absolute Neutrophil count** |  |  |  |  |  | NA/<0.001 |
| N | 44 | 20 | 15 | 60 | 0 |  |
| Mean (SD) | 4205.025 (2073.391) | 4950.764 (3453.647) | 5808.599 (2490.259) | 8946.773 (4912.114) | NA |  |
| Median | 3602.798 | 3933.506 | 5159.362 | 8188.342 | NA |  |
| IQR | 2563.947 | 3667.860 | 3996.700 | 7085.867 | NA |  |
| Q1, Q3 | 2570.976, 5134.924 | 2720.847, 6388.707 | 3671.150, 7667.851 | 4857.996, 11943.863 | NA |  |
| **Absolute Nutrophil count group** |  |  |  |  |  | NA/<0.001 |
| N-Miss | 0 | 0 | 0 | 0 | 454 |  |
| <8870 | 43 (97.7%) | 17 (85.0%) | 13 (86.7%) | 33 (55.0%) | 0 |  |
| >=8870 | 1 (2.3%) | 3 (15.0%) | 2 (13.3%) | 27 (45.0%) | 0 |  |
| **Absolute Monocyte** |  |  |  |  |  | NA/0.842 |
| N | 44 | 20 | 15 | 60 | 0 |  |
| Mean (SD) | 461.392 (201.834) | 505.119 (258.752) | 546.671 (291.601) | 527.265 (315.656) | NA |  |
| Median | 455.490 | 421.310 | 486.747 | 439.101 | NA |  |
| IQR | 255.058 | 125.432 | 179.822 | 332.093 | NA |  |
| Q1, Q3 | 320.128, 575.186 | 395.077, 520.508 | 382.922, 562.744 | 329.800, 661.893 | NA |  |
| **CD4/CD8 ratio** |  |  |  |  |  | 0.007/0.439 |
| N | 44 | 20 | 15 | 60 | 454 |  |
| Mean (SD) | 1.994 (2.027) | 1.477 (0.510) | 1.623 (0.907) | 1.672 (1.258) | 2.186 (1.844) |  |
| Median | 1.629 | 1.497 | 1.461 | 1.383 | 1.746 |  |
| IQR | 1.060 | 0.724 | 0.669 | 1.196 | 1.360 |  |
| Q1, Q3 | 1.133, 2.193 | 1.064, 1.788 | 1.237, 1.906 | 0.811, 2.007 | 1.209, 2.569 |  |
| **Neutrophil/Lymphocyte Ratio** |  |  |  |  |  | < 0.001/<0.001 |
| N | 44 | 20 | 15 | 60 | 454 |  |
| Mean (SD) | 2.462 (1.785) | 2.528 (2.295) | 2.681 (1.361) | 8.206 (6.329) | 1.841 (1.140) |  |
| Median | 1.885 | 1.540 | 2.238 | 5.941 | 1.572 |  |
| IQR | 1.281 | 1.772 | 1.068 | 9.250 | 1.054 |  |
| Q1, Q3 | 1.486, 2.767 | 1.246, 3.018 | 1.887, 2.955 | 2.750, 11.999 | 1.159, 2.213 |  |
| **Neutrophil/Lymphocyte Ratio group** |  |  |  |  |  | < 0.001/<0.001 |
| <4 | 41 (93.2%) | 17 (85.0%) | 14 (93.3%) | 19 (31.7%) | 438 (96.5%) |  |
| ≥4 | 3 (6.8%) | 3 (15.0%) | 1 (6.7%) | 41 (68.3%) | 16 (3.5%) |  |
| **Lymphocyte/Monocyte Ratio** |  |  |  |  |  | <0.001/<0.001 |
| N | 44 | 20 | 15 | 60 | 0 |  |
| Mean (SD) | 4.587 (1.482) | 5.182 (2.454) | 4.691 (1.618) | 3.401 (1.784) | NA |  |
| Median | 4.601 | 5.278 | 4.359 | 2.857 | NA |  |
| IQR | 1.576 | 2.541 | 2.194 | 2.692 | NA |  |
| Q1, Q3 | 3.729, 5.305 | 3.655, 6.196 | 3.782, 5.977 | 2.020, 4.712 | NA |  |
| **Total lymphocyte %** |  |  |  |  |  | < 0.001/<0.001 |
| N | 44 | 20 | 15 | 60 | 454 |  |
| Mean (SD) | 0.312 (0.093) | 0.336 (0.123) | 0.278 (0.067) | 0.174 (0.129) | 0.371 (0.109) |  |
| Median | 0.331 | 0.371 | 0.282 | 0.136 | 0.371 |  |
| IQR | 0.126 | 0.170 | 0.084 | 0.170 | 0.145 |  |
| Q1, Q3 | 0.247, 0.373 | 0.243, 0.413 | 0.240, 0.324 | 0.074, 0.244 | 0.294, 0.439 |  |

*Pearson Chi-sq for categorical variables, Kruskal-Wallis test for continuous factors; p value for comparisons including controls/p value for comparisons for cases only

**Supplemental Table 3**: Follow-up Pairwise Comparisons of pre-surgery Immune Cell Subset fractions (proportions) and absolute counts between WHO 2016 Classification groups; comparisons among Immune Profile Study (IPS) glioma patients only, as well as with Adult Glioma Study (AGS) controls

|  | **Comparisons without AGS controls** |  |
| --- | --- | --- |
| **Cell subset** | **Comparison** | **Unadj. p-value** |
| **%CD4 T cell** | IDHmt Astro – IDHmt 1p19q codel Oligo | 0.66 |
|  | IDHmt GBM - IDHmt 1p19q codel Oligo | 0.29 |
|  | IDHwt GBM – IDHmt 1p19q codel Oligo | 5.53e-07 |
|  | IDHmt GBM – IDHmt Astro | 0.20 |
|  | IDHwt GBM – IDHmt Astro | 1.68e-05 |
|  | IDHwt GBM – IDHmt GBM | 0.19 |
|  |  |  |
| **%CD8 T cell** | IDHmt Astro – IDHmt 1p19q codel Oligo | 0.39 |
|  | IDHmt GBM - IDHmt 1p19q codel Oligo | 0.64 |
|  | IDHwt GBM – IDHmt 1p19q codel Oligo | 5.95e-07 |
|  | IDHmt GBM – IDHmt Astro | 0.28 |
|  | IDHwt GBM – IDHmt Astro | 2.22e-06 |
|  | IDHwt GBM – IDHmt GBM | 0.0033 |
|  |  |  |
| **%Natural Killer cell** | IDHmt Astro – IDHmt 1p19q codel Oligo | 0.67 |
|  | IDHmt GBM - IDHmt 1p19q codel Oligo | 0.22 |
|  | IDHwt GBM – IDHmt 1p19q codel Oligo | 9.43e-07 |
|  | IDHmt GBM – IDHmt Astro | 0.095 |
|  | IDHwt GBM – IDHmt Astro | 0.0009 |
|  | IDHwt GBM – IDHmt GBM | 0.32 |
|  |  |  |
| **%B cells** | IDHmt Astro – IDHmt 1p19q codel Oligo | 0.30 |
|  | IDHmt GBM - IDHmt 1p19q codel Oligo | 0.35 |
|  | IDHwt GBM – IDHmt 1p19q codel Oligo | 9.37e-05 |
|  | IDHmt GBM – IDHmt Astro | 1.0 |
|  | IDHwt GBM – IDHmt Astro | 4.39e-05 |
|  | IDHwt GBM – IDHmt GBM | 0.00025 |
|  |  |  |
| **%Monocytes** | IDHmt Astro – IDHmt 1p19q codel Oligo | 0.70 |
|  | IDHmt GBM - IDHmt 1p19q codel Oligo | 0.365 |
|  | IDHwt GBM – IDHmt 1p19q codel Oligo | 0.00023 |
|  | IDHmt GBM – IDHmt Astro | 0.63 |
|  | IDHwt GBM – IDHmt Astro | 0.015 |
|  | IDHwt GBM – IDHmt GBM | 0.110 |
|  |  |  |
| **%Neutrophil** | IDHmt Astro – IDHmt 1p19q codel Oligo | 0.52 |
|  | IDHmt GBM - IDHmt 1p19q codel Oligo | 0.33 |
|  | IDHwt GBM – IDHmt 1p19q codel Oligo | 1.60e-07 |
|  | IDHmt GBM – IDHmt Astro | 0.176 |
|  | IDHwt GBM – IDHmt Astro | 2.62e-06 |
|  | IDHwt GBM – IDHmt GBM | 0.009 |
|  |  |  |
| **Total Cell Count** | IDHmt Astro – IDHmt 1p19q codel Oligo | 0.438 |
|  | IDHmt GBM - IDHmt 1p19q codel Oligo | 0.068 |
|  | IDHwt GBM – IDHmt 1p19q codel Oligo | 1.76e-05 |
|  | IDHmt GBM – IDHmt Astro | 0.326 |
|  | IDHwt GBM – IDHmt Astro | 0.0042 |
|  | IDHwt GBM – IDHmt GBM | 0.162 |
|  |  |  |
| **Absolute CD4 T cell** | IDHmt Astro – IDHmt 1p19q codel Oligo | 0.497 |
|  | IDHmt GBM - IDHmt 1p19q codel Oligo | 0.978 |
|  | IDHwt GBM – IDHmt 1p19q codel Oligo | 0.001 |
|  | IDHmt GBM – IDHmt Astro | 0.575 |
|  | IDHwt GBM – IDHmt Astro | 0.001 |
|  | IDHwt GBM – IDHmt GBM | 0.026 |
|  |  |  |
| **Absolute CD8 T cell** | IDHmt Astro – IDHmt 1p19q codel Oligo | 0.085 |
|  | IDHmt GBM - IDHmt 1p19q codel Oligo | 0.466 |
|  | IDHwt GBM – IDHmt 1p19q codel Oligo | 0.0058 |
|  | IDHmt GBM – IDHmt Astro | 0.470 |
|  | IDHwt GBM – IDHmt Astro | 8.77e-05 |
|  | IDHwt GBM – IDHmt GBM | 0.008 |
|  |  |  |
| **Absolute B cells** | IDHmt Astro – IDHmt 1p19q codel Oligo | 0.048 |
|  | IDHmt GBM - IDHmt 1p19q codel Oligo | 0.027 |
|  | IDHwt GBM – IDHmt 1p19q codel Oligo | 0.632 |
|  | IDHmt GBM – IDHmt Astro | 0.714 |
|  | IDHwt GBM – IDHmt Astro | 0.089 |
|  | IDHwt GBM – IDHmt GBM | 0.051 |
|  |  |  |
| **Absolute Lymphocyte Count** | IDHmt Astro – IDHmt 1p19q codel Oligo | 0.271 |
|  | IDHmt GBM - IDHmt 1p19q codel Oligo | 0.327 |
|  | IDHwt GBM – IDHmt 1p19q codel Oligo | 0.0045 |
|  | IDHmt GBM – IDHmt Astro | 0.991 |
|  | IDHwt GBM – IDHmt Astro | 0.0086 |
|  | IDHwt GBM – IDHmt GBM | 0.003 |
|  |  |  |
| **Absolute Neutrophil** | IDHmt Astro – IDHmt 1p19q codel Oligo | 0.690 |
|  | IDHmt GBM - IDHmt 1p19q codel Oligo | 0.083 |
|  | IDHwt GBM – IDHmt 1p19q codel Oligo | 1.20e-07 |
|  | IDHmt GBM – IDHmt Astro | 0.229 |
|  | IDHwt GBM – IDHmt Astro | 0.0003 |
|  | IDHwt GBM – IDHmt GBM | 0.065 |
|  |  |  |
| **Neutrophil/Lymphocyte Ratio** | IDHmt Astro – IDHmt 1p19q codel Oligo | 0.511 |
|  | IDHmt GBM - IDHmt 1p19q codel Oligo | 0.326 |
|  | IDHwt GBM – IDHmt 1p19q codel Oligo | 1.02e-07 |
|  | IDHmt GBM – IDHmt Astro | 0.168 |
|  | IDHwt GBM – IDHmt Astro | 1.77e-06 |
|  | IDHwt GBM – IDHmt GBM | 0.0082 |
|  |  |  |
| **Lymphocyte/Monocyte Ratio** | IDHmt Astro – IDHmt 1p19q codel Oligo | 0.592 |
|  | IDHmt GBM - IDHmt 1p19q codel Oligo | 0.989 |
|  | IDHwt GBM – IDHmt 1p19q codel Oligo | 0.0046 |
|  | IDHmt GBM – IDHmt Astro | 0.681 |
|  | IDHwt GBM – IDHmt Astro | 0.0011 |
|  | IDHwt GBM – IDHmt GBM | 0.0154 |
|  |  |  |
| **% Total Lymphocyte** | IDHmt Astro – IDHmt 1p19q codel Oligo | 0.506 |
|  | IDHmt GBM - IDHmt 1p19q codel Oligo | 0.342 |
|  | IDHwt GBM – IDHmt 1p19q codel Oligo | 7.25e-08 |
|  | IDHmt GBM – IDHmt Astro | 0.175 |
|  | IDHwt GBM – IDHmt Astro | 1.34e-06 |
|  | IDHwt GBM – IDHmt GBM | 0.0066 |
|  |  |  |
|  | **Comparisons with AGS Controls** |  |
| **%CD4 T cell** | IDHmt Astro – IDHmt 1p19q codel Oligo | 0.52 |
|  | IDHmt GBM - IDHmt 1p19q codel Oligo | 0.22 |
|  | IDHwt GBM – IDHmt 1p19q codel Oligo | 1.47e-05 |
|  | AGS controls – IDHmt 1p19q codel Oligo | 0.010 |
|  | IDHmt GBM – IDHmt Astro | 0.114 |
|  | IDHwt GBM – IDHmt Astro | 6.29e-05 |
|  | AGS controls – IDHmt Astro | 0.31 |
|  | IDHwt GBM – IDHmt GBM | 0.087 |
|  | AGS controls – IDHmt GBM | 0.003 |
|  | AGS controls – IDHwt GBM | <2.22e-16 |
|  |  |  |
| **%CD8 T cell** | IDHmt Astro – IDHmt 1p19q codel Oligo | 0.31 |
|  | IDHmt GBM - IDHmt 1p19q codel Oligo | 0.56 |
|  | IDHwt GBM – IDHmt 1p19q codel Oligo | 9.39e-06 |
|  | AGS controls – IDHmt 1p19q codel Oligo | 0.08 |
|  | IDHmt GBM – IDHmt Astro | 0.19 |
|  | IDHwt GBM – IDHmt Astro | 8.15e-05 |
|  | AGS controls – IDHmt Astro | 1.0 |
|  | IDHwt GBM – IDHmt GBM | 0.015 |
|  | AGS controls – IDHmt GBM | 0.087 |
|  | AGS controls – IDHwt GBM | <2.22e-16 |
|  |  |  |
| **%Natural Killer cell** | IDHmt Astro – IDHmt 1p19q codel Oligo | 0.67 |
|  | IDHmt GBM - IDHmt 1p19q codel Oligo | 0.012 |
|  | IDHwt GBM – IDHmt 1p19q codel Oligo | 3.33e-07 |
|  | AGS controls – IDHmt 1p19q codel Oligo | 0.20 |
|  | IDHmt GBM – IDHmt Astro | 0.063 |
|  | IDHwt GBM – IDHmt Astro | 0.0005 |
|  | AGS controls – IDHmt Astro | 0.70 |
|  | IDHwt GBM – IDHmt GBM | 0.36 |
|  | AGS controls – IDHmt GBM | 0.037 |
|  | AGS controls – IDHwt GBM | 3.74e-09 |
|  |  |  |
| **%B cells** | IDHmt Astro – IDHmt 1p19q codel Oligo | 0.31 |
|  | IDHmt GBM - IDHmt 1p19q codel Oligo | 0.34 |
|  | IDHwt GBM – IDHmt 1p19q codel Oligo | 0.0007 |
|  | AGS controls – IDHmt 1p19q codel Oligo | 0.0042 |
|  | IDHmt GBM – IDHmt Astro | 0.97 |
|  | IDHwt GBM – IDHmt Astro | 0.0002 |
|  | AGS controls – IDHmt Astro | 0.43 |
|  | IDHwt GBM – IDHmt GBM | 0.0009 |
|  | AGS controls – IDHmt GBM | 0.52 |
|  | AGS controls – IDHwt GBM | 2.78e-16 |
|  |  |  |
| **%Monocytes** | IDHmt Astro – IDHmt 1p19q codel Oligo | 0.75 |
|  | IDHmt GBM - IDHmt 1p19q codel Oligo | 0.36 |
|  | IDHwt GBM – IDHmt 1p19q codel Oligo | 0.0006 |
|  | AGS controls – IDHmt 1p19q codel Oligo | 0.31 |
|  | IDHmt GBM – IDHmt Astro | 0.58 |
|  | IDHwt GBM – IDHmt Astro | 0.022 |
|  | AGS controls – IDHmt Astro | 0.28 |
|  | IDHwt GBM – IDHmt GBM | 0.16 |
|  | AGS controls – IDHmt GBM | 0.098 |
|  | AGS controls – IDHwt GBM | 9.74e-10 |
|  |  |  |
| **%Neutrophil** | IDHmt Astro – IDHmt 1p19q codel Oligo | 0.32 |
|  | IDHmt GBM - IDHmt 1p19q codel Oligo | 0.18 |
|  | IDHwt GBM – IDHmt 1p19q codel Oligo | 1.72e-05 |
|  | AGS controls – IDHmt 1p19q codel Oligo | 0.014 |
|  | IDHmt GBM – IDHmt Astro | 0.05 |
|  | IDHwt GBM – IDHmt Astro | 1.36e-05 |
|  | AGS controls – IDHmt Astro | 0.60 |
|  | IDHwt GBM – IDHmt GBM | 0.116 |
|  | AGS controls – IDHmt GBM | 0.0027 |
|  | AGS controls – IDHwt GBM | <2.22e-16 |
|  |  |  |
| **% Total Lymphocytes** | IDHmt Astro – IDHmt 1p19q codel Oligo | 0.27 |
|  | IDHmt GBM - IDHmt 1p19q codel Oligo | 0.21 |
|  | IDHwt GBM – IDHmt 1p19q codel Oligo | 3.08e-05 |
|  | AGS controls – IDHmt 1p19q codel Oligo | 0.002 |
|  | IDHmt GBM – IDHmt Astro | 0.05 |
|  | IDHwt GBM – IDHmt Astro | 1.38e-05 |
|  | AGS controls – IDHmt Astro | 0.40 |
|  | IDHwt GBM – IDHmt GBM | 0.11 |
|  | AGS controls – IDHmt GBM | 0.001 |
|  | AGS controls – IDHwt GBM | <2.22e-16 |
|  |  |  |
| **CD4/CD8 ratio** | IDHmt Astro – IDHmt 1p19q codel Oligo | 0.30 |
|  | IDHmt GBM - IDHmt 1p19q codel Oligo | 0.59 |
|  | IDHwt GBM – IDHmt 1p19q codel Oligo | 0.16 |
|  | AGS controls – IDHmt 1p19q codel Oligo | 0.33 |
|  | IDHmt GBM – IDHmt Astro | 0.73 |
|  | IDHwt GBM – IDHmt Astro | 0.99 |
|  | AGS controls – IDHmt Astro | 0.057 |
|  | IDHwt GBM – IDHmt GBM | 0.68 |
|  | AGS controls – IDHmt GBM | 0.23 |
|  | AGS controls – IDHwt GBM | 0.0016 |
|  |  |  |
| **Neutrophil/Lymphocyte Ratio** | IDHmt Astro – IDHmt 1p19q codel Oligo | 0.27 |
|  | IDHmt GBM - IDHmt 1p19q codel Oligo | 0.19 |
|  | IDHwt GBM – IDHmt 1p19q codel Oligo | 2.62e-05 |
|  | AGS controls – IDHmt 1p19q codel Oligo | 0.004 |
|  | IDHmt GBM – IDHmt Astro | 0.043 |
|  | IDHwt GBM – IDHmt Astro | 1.12e-05 |
|  | AGS controls – IDHmt Astro | 0.50 |
|  | IDHwt GBM – IDHmt GBM | 0.12 |
|  | AGS controls – IDHmt GBM | 0.0013 |
|  | AGS controls – IDHwt GBM | <2.22e-16 |
|  |  |  |
| **Lymphocyte/Monocyte Ratio** | IDHmt Astro – IDHmt 1p19q codel Oligo | 0.47 |
|  | IDHmt GBM - IDHmt 1p19q codel Oligo | 0.97 |
|  | IDHwt GBM – IDHmt 1p19q codel Oligo | 0.0007 |
|  | AGS controls – IDHmt 1p19q codel Oligo | 0.21 |
|  | IDHmt GBM – IDHmt Astro | 0.54 |
|  | IDHwt GBM – IDHmt Astro | 0.0007 |
|  | AGS controls – IDHmt Astro | 1.0 |
|  | IDHwt GBM – IDHmt GBM | 0.021 |
|  | AGS controls – IDHmt GBM | 0.43 |
|  | AGS controls – IDHwt GBM | 2.29e-10 |

**Supplemental Table 4** Participant Characteristics and Pre-surgery Immune Cell Profiles for Immune Profile Study (IPS) Glioma Participants by Dexamethasone Use at Blood Draw and Adult Glioma Study (AGS) Controls (No Dex)

|  | AGS Control/No Dex (N=447) | Glioma/No Dex at Blood draw (N=76) | Glioma/Dex at Blood draw (N=63) | p value |
| --- | --- | --- | --- | --- |
| **Gender** |  |  |  | 0.320 (1) |
| F | 206 (46.1%) | 30 (39.5%) | 24 (38.1%) |  |
| M | 241 (53.9%) | 46 (60.5%) | 39 (61.9%) |  |
| **White race** |  |  |  | < 0.001 (1) |
| non-white | 129 (28.9%) | 10 (13.2%) | 4 (6.3%) |  |
| white | 318 (71.1%) | 66 (86.8%) | 59 (93.7%) |  |
| **Dexamethasone use in past mo** |  |  |  |  |
| No | 447 (100%) | 68 (89.5%) | 0 (0.0%) |  |
| Yes | 0 | 8 (10.5%) | 63 (100.0%) |  |
| **Tumor grade** |  |  |  | < 0.001 (1) |
| 2 | 0 | 30 (39.5%) | 5 (7.9%) |  |
| 3 | 0 | 21 (27.6%) | 6 (9.5%) |  |
| 4 | 0 | 25 (32.9%) | 52 (82.5%) |  |
| **WHO 2016 classification** |  |  |  | < 0.001 (1) |
| IDHmt 1p19q codel oligo | 0 | 38 (50.0%) | 6 (9.5%) |  |
| IDHmt Astro | 0 | 15 (19.7%) | 5 (7.9%) |  |
| IDHmt GBM | 0 | 8 (10.5%) | 7 (11.1%) |  |
| IDHwt GBM | 0 | 15 (19.7%) | 45 (71.4%) |  |
| **Diagnosis group** |  |  |  | < 0.001 (1) |
| new gbm | 0 (0.0%) | 18 (23.7%) | 49 (77.8%) |  |
| new lgg | 0 (0.0%) | 21 (27.6%) | 8 (12.7%) |  |
| rec lgg, still lgg | 0 (0.0%) | 30 (39.5%) | 3 (4.8%) |  |
| rec lgg, now gbm | 0 (0.0%) | 7 (9.2%) | 3 (4.8%) |  |
| control | 447 (100.0%) | 0 (0.0%) | 0 (0.0%) |  |
| **Age 1st Diagnosis** |  |  |  | 0.80 (2) |
| N | 0 | 42 | 15 |  |
| Mean (SD) | NA | 37.786 (10.584) | 42.400 (20.156) |  |
| Median | NA | 36.000 | 35.000 |  |
| IQR | NA | 13.000 | 35.000 |  |
| Q1, Q3 | NA | 30.750, 43.750 | 24.500, 59.500 |  |
| **Age at enrollment** |  |  |  | 0.007 (2) |
| N | 447 | 76 | 63 |  |
| Mean (SD) | 51.622 (15.327) | 47.368 (13.085) | 54.365 (17.143) |  |
| Median | 52.000 | 45.000 | 60.000 |  |
| IQR | 22.000 | 17.500 | 27.000 |  |
| Q1, Q3 | 41.000, 63.000 | 37.750, 55.250 | 40.000, 67.000 |  |
| **Natural Killer cell %** |  |  |  | < 0.001 (2) |
| N | 447 | 76 | 63 |  |
| Mean (SD) | 0.051 (0.026) | 0.056 (0.022) | 0.028 (0.023) |  |
| Median | 0.047 | 0.054 | 0.026 |  |
| IQR | 0.028 | 0.021 | 0.021 |  |
| Q1, Q3 | 0.034, 0.062 | 0.042, 0.063 | 0.013, 0.034 |  |
| **CD4 T cell %** |  |  |  | < 0.001 (2) |
| N | 447 | 76 | 63 |  |
| Mean (SD) | 0.161 (0.063) | 0.147 (0.050) | 0.046 (0.037) |  |
| Median | 0.157 | 0.151 | 0.034 |  |
| IQR | 0.080 | 0.073 | 0.046 |  |
| Q1, Q3 | 0.117, 0.197 | 0.111, 0.185 | 0.019, 0.065 |  |
| **CD8 T cell %** |  |  |  | < 0.001 (2) |
| N | 447 | 76 | 63 |  |
| Mean (SD) | 0.100 (0.056) | 0.090 (0.040) | 0.038 (0.030) |  |
| Median | 0.089 | 0.088 | 0.027 |  |
| IQR | 0.065 | 0.058 | 0.035 |  |
| Q1, Q3 | 0.061, 0.126 | 0.061, 0.119 | 0.017, 0.051 |  |
| **B cell %** |  |  |  | < 0.001 (2) |
| N | 447 | 76 | 63 |  |
| Mean (SD) | 0.058 (0.029) | 0.050 (0.021) | 0.032 (0.022) |  |
| Median | 0.053 | 0.046 | 0.027 |  |
| IQR | 0.030 | 0.028 | 0.022 |  |
| Q1, Q3 | 0.040, 0.070 | 0.034, 0.062 | 0.016, 0.039 |  |
| **Monocyte %** |  |  |  | < 0.001 (2) |
| N | 447 | 76 | 63 |  |
| Mean (SD) | 0.078 (0.026) | 0.076 (0.024) | 0.049 (0.025) |  |
| Median | 0.074 | 0.073 | 0.047 |  |
| IQR | 0.034 | 0.030 | 0.035 |  |
| Q1, Q3 | 0.059, 0.094 | 0.061, 0.090 | 0.030, 0.065 |  |
| **Neutrophil %** |  |  |  | < 0.001 (2) |
| N | 447 | 76 | 63 |  |
| Mean (SD) | 0.580 (0.113) | 0.594 (0.098) | 0.821 (0.098) |  |
| Median | 0.579 | 0.590 | 0.837 |  |
| IQR | 0.146 | 0.116 | 0.141 |  |
| Q1, Q3 | 0.510, 0.656 | 0.532, 0.648 | 0.758, 0.899 |  |
| **Total Cell Count** |  |  |  | < 0.001 (2) |
| N | 0 | 76 | 63 |  |
| Mean (SD) | NA | 6437.274 (2543.902) | 11609.297 (4523.465) |  |
| Median | NA | 5878.267 | 11320.000 |  |
| IQR | NA | 3341.250 | 5779.333 |  |
| Q1, Q3 | NA | 4469.000, 7810.250 | 8525.667, 14305.000 |  |
| **Total Cells Grouped** |  |  |  | < 0.001 (1) |
| <10K | 0 | 68 (89.5%) | 28 (44.4%) |  |
| >=10K | 0 | 8 (10.5%) | 35 (55.6%) |  |
| **Absolute CD4 cell count** |  |  |  | < 0.001 (2) |
| N | 0 | 76 | 63 |  |
| Mean (SD) | NA | 917.040 (448.136) | 505.432 (421.205) |  |
| Median | NA | 846.298 | 394.948 |  |
| IQR | NA | 501.022 | 510.072 |  |
| Q1, Q3 | NA | 599.192, 1100.215 | 185.296, 695.368 |  |
| **CD4 T cell group** |  |  |  | < 0.001 (1) |
| <500 | 0 | 11 (14.5%) | 40 (63.5%) |  |
| >=500 | 0 | 65 (85.5%) | 23 (36.5%) |  |
| **CD4 T cell group** |  |  |  | < 0.001 (1) |
| <200 | 0 | 1 (1.3%) | 18 (28.6%) |  |
| >=200 | 0 | 75 (98.7%) | 45 (71.4%) |  |
| **Absolute CD8 cell count** |  |  |  | < 0.001 (2) |
| N | 0 | 76 | 63 |  |
| Mean (SD) | NA | 541.030 (269.071) | 403.740 (321.899) |  |
| Median | NA | 507.733 | 270.941 |  |
| IQR | NA | 336.348 | 375.986 |  |
| Q1, Q3 | NA | 325.163, 661.512 | 176.413, 552.399 |  |
| **Absolute B cell count** |  |  |  | 0.48 (2) |
| N | 0 | 76 | 63 |  |
| Mean (SD) | NA | 314.093 (194.365) | 347.053 (234.351) |  |
| Median | NA | 271.480 | 291.806 |  |
| IQR | NA | 163.112 | 234.881 |  |
| Q1, Q3 | NA | 197.782, 360.893 | 190.409, 425.289 |  |
| **Absolute Natural Killer cell count** |  |  |  | < 0.006 (2) |
| N | 0 | 76 | 63 |  |
| Mean (SD) | NA | 349.692 (180.641) | 289.692 (223.651) |  |
| Median | NA | 317.777 | 232.539 |  |
| IQR | NA | 204.697 | 163.795 |  |
| Q1, Q3 | NA | 212.696, 417.393 | 174.014, 337.808 |  |
| **Absolute Lymphcyte count** |  |  |  | < 0.001 (2) |
| N | 0 | 76 | 63 |  |
| Mean (SD) | NA | 2121.855 (840.916) | 1545.917 (930.780) |  |
| Median | NA | 2048.633 | 1212.794 |  |
| IQR | NA | 1068.020 | 1043.846 |  |
| Q1, Q3 | NA | 1481.812, 2549.832 | 859.198, 1903.044 |  |
| ALC group |  |  |  | < 0.001 (1) |
| <1K | 0 | 4 (5.3%) | 24 (38.1%) |  |
| >=1K | 0 | 72 (94.7%) | 39 (61.9%) |  |
| **Absolute Neutrophil count** |  |  |  | < 0.001 (2) |
| N | 0 | 76 | 63 |  |
| Mean (SD) | NA | 3927.686 (1961.010) | 9674.089 (4291.803) |  |
| Median | NA | 3377.456 | 8641.751 |  |
| IQR | NA | 2200.110 | 4956.519 |  |
| Q1, Q3 | NA | 2408.240, 4608.350 | 7001.355, 11957.874 |  |
| **Absolute Neutrophil Count group** |  |  |  | < 0.001 (1) |
| <8870 | 0 | 73 (96.1%) | 33 (52.4%) |  |
| >=8870 | 0 | 3 (3.9%) | 30 (47.6%) |  |
| **Absolute Monocyte** |  |  |  | 0.50 (2) |
| N | 0 | 76 | 63 |  |
| Mean (SD) | NA | 469.646 (192.850) | 548.357 (341.477) |  |
| Median | NA | 437.041 | 492.268 |  |
| IQR | NA | 206.597 | 447.599 |  |
| Q1, Q3 | NA | 348.715, 555.311 | 310.147, 757.745 |  |
| **CD4/CD8 ratio** |  |  |  | < 0.001 (2) |
| N | 447 | 76 | 63 |  |
| Mean (SD) | 2.188 (1.854) | 2.001 (1.722) | 1.426 (0.964) |  |
| Median | 1.745 | 1.623 | 1.179 |  |
| IQR | 1.353 | 0.917 | 0.977 |  |
| Q1, Q3 | 1.210, 2.563 | 1.263, 2.181 | 0.738, 1.715 |  |
| **Neutrophil/Lymphocyte Ratio** |  |  |  | < 0.001 (2) |
| N | 447 | 76 | 63 |  |
| Mean (SD) | 1.851 (1.143) | 2.018 (1.217) | 8.541 (5.892) |  |
| Median | 1.589 | 1.721 | 6.727 |  |
| IQR | 1.058 | 1.045 | 8.057 |  |
| Q1, Q3 | 1.159, 2.217 | 1.296, 2.340 | 3.721, 11.778 |  |
| **Neutrophil/Lymphocyte Ratio grouped** |  |  |  | < 0.001 (1) |
| <4 | 431 (96.4%) | 73 (96.1%) | 18 (28.6%) |  |
| ≥4 | 16 (3.6%) | 3 (3.9%) | 45 (71.4%) |  |
| **Lymphocyte/Monocyte Ratio** |  |  |  | < 0.001 (2) |
| N | 447 | 76 | 63 |  |
| Mean (SD) | 5.182 (2.155) | 4.798 (1.665) | 3.417 (1.914) |  |
| Median | 4.792 | 4.672 | 2.846 |  |
| IQR | 2.631 | 2.152 | 2.671 |  |
| Q1, Q3 | 3.743, 6.374 | 3.729, 5.881 | 1.788, 4.459 |  |
| **Total lymphocyte %** |  |  |  | < 0.001 (2) |
| N | 447 | 76 | 63 |  |
| Mean (SD) | 0.369 (0.107) | 0.342 (0.091) | 0.144 (0.082) |  |
| Median | 0.370 | 0.345 | 0.127 |  |
| IQR | 0.145 | 0.117 | 0.129 |  |
| Q1, Q3 | 0.294, 0.439 | 0.285, 0.402 | 0.074, 0.203 |  |

1. Pearson’s Chi-squared test
2. Kruskal-Wallis rank sum test

**Supplemental Table 5** Dunn Test Pairwise Comparison of Immune Profile Study (IPS) Glioma Patients and Adult Glioma Study (AGS) Controls by Dexamethasone use at blood draw

| **Immune Cell Subset** | **Comparison Groups** | **Unadjusted p-value** |
| --- | --- | --- |
| **%CD4 T cells** | Glioma/No Dex – AGS control/No Dex | 0.23 |
|  | Glioma/Dex – AGS control/No Dex | <2e-16 |
|  | Glioma/ Dex – Glioma/No Dex | <2e-16 |
|  |  |  |
| **%CD8 T cells** | Glioma/No Dex – AGS control/No Dex | 0.44 |
|  | Glioma/Dex – AGS control/No Dex | <2.22e-16 |
|  | Glioma/ Dex – Glioma/No Dex | 3.19e-16 |
|  |  |  |
| **%Natural Killer cells** | Glioma/No Dex – AGS control/No Dex | 0.02 |
|  | Glioma/Dex – AGS control/No Dex | 8.44e-15 |
|  | Glioma/ Dex – Glioma/No Dex | 5.15e-15 |
|  |  |  |
| **%B cells** | Glioma/No Dex – AGS control/No Dex | 0.02 |
|  | Glioma/Dex – AGS control/No Dex | <2.22e-16 |
|  | Glioma/ Dex – Glioma/No Dex | 1.54e-06 |
|  |  |  |
| **%Monocytes** | Glioma/No Dex – AGS control/No Dex | 0.78 |
|  | Glioma/Dex – AGS control/No Dex | 9.03e-15 |
|  | Glioma/ Dex – Glioma/No Dex | 3.13e-09 |
|  |  |  |
| **%Neutrophils** | Glioma/No Dex – AGS control/No Dex | 0.43 |
|  | Glioma/Dex – AGS control/No Dex | <2e-16 |
|  | Glioma/ Dex – Glioma/No Dex | <2e-16 |
|  |  |  |
| **% Total Lymphocytes** | Glioma/No Dex – AGS control/No Dex | 0.08 |
|  | Glioma/ Dex – AGS control/No Dex | <2.22e-16 |
|  | Glioma/ Dex – Glioma/No Dex | 1.13e-15 |
|  |  |  |
| **CD4/CD8 Ratio** | Glioma/No Dex – AGS control/No Dex | 0.42 |
|  | Glioma/ Dex – AGS control/No Dex | 6.16e-06 |
|  | Glioma/ Dex – Glioma/No Dex | 0.003 |
|  |  |  |
| **Neutrophil/Lymphocyte Ratio** | Glioma/No Dex – AGS control/No Dex | 0.50 |
|  | Glioma/Dex – AGS control/No Dex | <2.22e-16 |
|  | Glioma/ Dex – Glioma/No Dex | 5.04e-16 |

**Supplemental Table 6**. Comparison of Participant Characteristics and Pre-surgery Immune Cell subset fractions (proportions) and absolute count; By Dexamethasone use at Blood draw for Immune Profile Study (IPS) glioma patients by WHO 2016 classification and Adult Glioma Study (AGS) controls

|  | no Dex-IDHmut 1p19qdel oligo (N=38) | Dex-IDHmut 1p19qdel oligo (N=6) | no Dex-IDHmut Astro (N=15) | Dex-IDHmut Astro (N=5) | no Dex-IDHmut GBM (N=8) | Dex-IDHmut GBM (N=7) | no Dex-IDHwt GBM (N=15) | Dex-IDHwt GBM (N=45) | 9 AGS cntrl (N=454) | p value  */** |
| --- | --- | --- | --- | --- | --- | --- | --- | --- | --- | --- |
| **Gender** |  |  |  |  |  |  |  |  |  | 0.379/0.326 |
| F | 16 (42.1%) | 4 (66.7%) | 6 (40.0%) | 0 (0.0%) | 3 (37.5%) | 1 (14.3%) | 5 (33.3%) | 19 (42.2%) | 207 (45.6%) |  |
| M | 22 (57.9%) | 2 (33.3%) | 9 (60.0%) | 5 (100.0%) | 5 (62.5%) | 6 (85.7%) | 10 (66.7%) | 26 (57.8%) | 247 (54.4%) |  |
| **White** |  |  |  |  |  |  |  |  |  | 0.548/0.003 |
| non-white | 6 (15.8%) | 1 (16.7%) | 1 (6.7%) | 0 (0.0%) | 2 (25.0%) | 0 (0.0%) | 1 (6.7%) | 3 (6.7%) | 132 (29.1%) |  |
| white | 32 (84.2%) | 5 (83.3%) | 14 (93.3%) | 5 (100.0%) | 6 (75.0%) | 7 (100.0%) | 14 (93.3%) | 42 (93.3%) | 322 (70.9%) |  |
| **Tumor grade** |  |  |  |  |  |  |  |  |  |  |
| N-Miss | 0 | 0 | 0 | 0 | 0 | 0 | 0 | 0 | 454 |  |
| 2 | 20 (52.6%) | 3 (50.0%) | 10 (66.7%) | 2 (40.0%) | 0 (0.0%) | 0 (0.0%) | 0 (0.0%) | 0 (0.0%) | 0 |  |
| 3 | 16 (42.1%) | 3 (50.0%) | 5 (33.3%) | 3 (60.0%) | 0 (0.0%) | 0 (0.0%) | 0 (0.0%) | 0 (0.0%) | 0 |  |
| 4 | 2 (5.3%) | 0 (0.0%) | 0 (0.0%) | 0 (0.0%) | 8 (100.0%) | 7 (100.0%) | 15 (100.0%) | 45 (100.0%) | 0 |  |
| **Diagnosis group** |  |  |  |  |  |  |  |  |  | <0.001/< 0.001 |
| 1 new gbm | 0 (0.0%) | 0 (0.0%) | 0 (0.0%) | 0 (0.0%) | 3 (37.5%) | 5 (71.4%) | 15 (100.0%) | 44 (97.8%) | 0 (0.0%) |  |
| 2 new lgg | 14 (36.8%) | 3 (50.0%) | 7 (46.7%) | 5 (100.0%) | 0 (0.0%) | 0 (0.0%) | 0 (0.0%) | 0 (0.0%) | 0 (0.0%) |  |
| 3 rec lgg, still lgg | 22 (57.9%) | 3 (50.0%) | 8 (53.3%) | 0 (0.0%) | 0 (0.0%) | 0 (0.0%) | 0 (0.0%) | 0 (0.0%) | 0 (0.0%) |  |
| 4 rec lgg, now gbm | 2 (5.3%) | 0 (0.0%) | 0 (0.0%) | 0 (0.0%) | 5 (62.5%) | 2 (28.6%) | 0 (0.0%) | 1 (2.2%) | 0 (0.0%) |  |
| 5 control | 0 (0.0%) | 0 (0.0%) | 0 (0.0%) | 0 (0.0%) | 0 (0.0%) | 0 (0.0%) | 0 (0.0%) | 0 (0.0%) | 454 (100.0%) |  |
| **Age 1st Diagnosis** |  |  |  |  |  |  |  |  |  |  |
| N | 26 | 3 | 9 | 0 | 6 | 3 | 1 | 9 | 0 |  |
| Mean (SD) | 37.846 (7.908) | 30.667 (12.014) | 31.778 (7.513) | NA | 40.833 (14.331) | 23.667 (2.887) | 72.000 (NA) | 52.556 (19.327) | NA |  |
| Median | 36.500 | 30.000 | 30.000 | NA | 46.000 | 22.000 | 72.000 | 58.000 | NA |  |
| IQR | 8.750 | 12.000 | 9.000 | NA | 16.500 | 2.500 | 0.000 | 31.000 | NA |  |
| Q1, Q3 | 34.000, 42.750 | 24.500, 36.500 | 27.000, 36.000 | NA | 30.250, 46.750 | 22.000, 24.500 | 72.000, 72.000 | 35.000, 66.000 | NA |  |
| **Age at enrollment** |  |  |  |  |  |  |  |  |  | <0.001/< 0.001 |
| N | 38 | 6 | 15 | 5 | 8 | 7 | 15 | 45 | 454 |  |
| Mean (SD) | 46.053 (9.174) | 45.000 (14.765) | 38.467 (9.657) | 33.000 (8.396) | 45.500 (12.444) | 31.143 (9.424) | 60.600 (15.652) | 61.600 (12.948) | 51.773 (15.527) |  |
| Median | 46.000 | 45.500 | 37.000 | 31.000 | 44.000 | 27.000 | 65.000 | 64.000 | 52.000 |  |
| IQR | 15.750 | 22.250 | 7.000 | 9.000 | 19.250 | 6.500 | 21.000 | 11.000 | 22.000 |  |
| Q1, Q3 | 38.000, 53.750 | 34.750, 57.000 | 32.000, 39.000 | 31.000, 40.000 | 36.750, 56.000 | 25.500, 32.000 | 48.500, 69.500 | 58.000, 69.000 | 41.000, 63.000 |  |
| **Natural Killer cell %** |  |  |  |  |  |  |  |  |  | <0.001/< 0.001 |
| N | 38 | 6 | 15 | 5 | 8 | 7 | 15 | 45 | 454 |  |
| Mean (SD) | 0.055 (0.020) | 0.050 (0.042) | 0.060 (0.019) | 0.025 (0.015) | 0.041 (0.019) | 0.043 (0.044) | 0.061 (0.029) | 0.024 (0.013) | 0.051 (0.028) |  |
| Median | 0.055 | 0.035 | 0.057 | 0.021 | 0.040 | 0.029 | 0.056 | 0.022 | 0.047 |  |
| IQR | 0.017 | 0.059 | 0.022 | 0.025 | 0.027 | 0.008 | 0.036 | 0.020 | 0.028 |  |
| Q1, Q3 | 0.046, 0.063 | 0.019, 0.078 | 0.050, 0.072 | 0.013, 0.038 | 0.026, 0.054 | 0.026, 0.034 | 0.041, 0.077 | 0.012, 0.033 | 0.034, 0.062 |  |
| **CD4 T cell %** |  |  |  |  |  |  |  |  |  | <0.001/< 0.001 |
| N | 38 | 6 | 15 | 5 | 8 | 7 | 15 | 45 | 454 |  |
| Mean (SD) | 0.141 (0.049) | 0.064 (0.053) | 0.166 (0.042) | 0.054 (0.036) | 0.141 (0.044) | 0.067 (0.043) | 0.145 (0.061) | 0.039 (0.033) | 0.161 (0.064) |  |
| Median | 0.142 | 0.036 | 0.179 | 0.047 | 0.152 | 0.052 | 0.154 | 0.028 | 0.157 |  |
| IQR | 0.066 | 0.066 | 0.053 | 0.009 | 0.075 | 0.039 | 0.064 | 0.050 | 0.080 |  |
| Q1, Q3 | 0.108, 0.174 | 0.029, 0.094 | 0.139, 0.192 | 0.043, 0.052 | 0.100, 0.174 | 0.040, 0.078 | 0.120, 0.184 | 0.010, 0.060 | 0.118, 0.197 |  |
| **CD8 T cell %** |  |  |  |  |  |  |  |  |  | <0.001/< 0.001 |
| N | 38 | 6 | 15 | 5 | 8 | 7 | 15 | 45 | 454 |  |
| Mean (SD) | 0.085 (0.036) | 0.058 (0.036) | 0.107 (0.032) | 0.050 (0.023) | 0.080 (0.029) | 0.068 (0.042) | 0.090 (0.056) | 0.029 (0.023) | 0.100 (0.057) |  |
| Median | 0.081 | 0.048 | 0.110 | 0.044 | 0.079 | 0.069 | 0.082 | 0.020 | 0.089 |  |
| IQR | 0.058 | 0.037 | 0.035 | 0.042 | 0.038 | 0.035 | 0.073 | 0.020 | 0.066 |  |
| Q1, Q3 | 0.056, 0.114 | 0.033, 0.070 | 0.086, 0.120 | 0.030, 0.072 | 0.057, 0.095 | 0.041, 0.076 | 0.050, 0.123 | 0.014, 0.034 | 0.061, 0.126 |  |
| **Bcell %** |  |  |  |  |  |  |  |  |  | <0.001/< 0.001 |
| N | 38 | 6 | 15 | 5 | 8 | 7 | 15 | 45 | 454 |  |
| Mean (SD) | 0.047 (0.020) | 0.036 (0.018) | 0.058 (0.025) | 0.040 (0.014) | 0.049 (0.012) | 0.062 (0.041) | 0.048 (0.024) | 0.026 (0.014) | 0.058 (0.029) |  |
| Median | 0.046 | 0.033 | 0.047 | 0.039 | 0.047 | 0.040 | 0.041 | 0.023 | 0.053 |  |
| IQR | 0.028 | 0.022 | 0.031 | 0.005 | 0.015 | 0.044 | 0.028 | 0.017 | 0.030 |  |
| Q1, Q3 | 0.029, 0.057 | 0.028, 0.050 | 0.042, 0.073 | 0.034, 0.039 | 0.042, 0.058 | 0.036, 0.079 | 0.029, 0.058 | 0.016, 0.033 | 0.040, 0.070 |  |
| **Monocyte %** |  |  |  |  |  |  |  |  |  | <0.001/< 0.001 |
| N | 38 | 6 | 15 | 5 | 8 | 7 | 15 | 45 | 454 |  |
| Mean (SD) | 0.075 (0.022) | 0.061 (0.037) | 0.073 (0.025) | 0.065 (0.023) | 0.081 (0.022) | 0.049 (0.018) | 0.081 (0.029) | 0.045 (0.024) | 0.078 (0.027) |  |
| Median | 0.071 | 0.053 | 0.071 | 0.070 | 0.079 | 0.049 | 0.083 | 0.039 | 0.074 |  |
| IQR | 0.032 | 0.022 | 0.017 | 0.030 | 0.023 | 0.017 | 0.032 | 0.034 | 0.034 |  |
| Q1, Q3 | 0.059, 0.090 | 0.049, 0.071 | 0.061, 0.078 | 0.053, 0.083 | 0.070, 0.093 | 0.042, 0.059 | 0.063, 0.096 | 0.028, 0.063 | 0.060, 0.094 |  |
| **Neutrophil %** |  |  |  |  |  |  |  |  |  | <0.001/< 0.001 |
| N | 38 | 6 | 15 | 5 | 8 | 7 | 15 | 45 | 454 |  |
| Mean (SD) | 0.609 (0.089) | 0.741 (0.105) | 0.552 (0.084) | 0.780 (0.084) | 0.620 (0.031) | 0.723 (0.084) | 0.586 (0.141) | 0.851 (0.085) | 0.578 (0.116) |  |
| Median | 0.605 | 0.719 | 0.533 | 0.799 | 0.626 | 0.711 | 0.560 | 0.870 | 0.579 |  |
| IQR | 0.105 | 0.148 | 0.092 | 0.086 | 0.033 | 0.078 | 0.173 | 0.120 | 0.145 |  |
| Q1, Q3 | 0.556, 0.661 | 0.666, 0.815 | 0.489, 0.581 | 0.756, 0.842 | 0.601, 0.634 | 0.676, 0.754 | 0.501, 0.674 | 0.792, 0.912 | 0.510, 0.655 |  |
| **Total Cell Count** |  |  |  |  |  |  |  |  |  |  |
| N | 38 | 6 | 15 | 5 | 8 | 7 | 15 | 45 | 0 | <0.001 (2)/NA |
| Mean (SD) | 6508.335 (2573.564) | 6896.644 (3294.400) | 5885.153 (1800.399) | 12455.867 (3373.451) | 7456.375 (3948.617) | 10012.981 (2714.231) | 6265.851 (2271.929) | 12391.903 (4627.984) | NA |  |
| Median | 6143.167 | 7321.750 | 5659.667 | 12416.667 | 5961.000 | 10700.000 | 5860.000 | 11833.333 | NA |  |
| IQR | 3864.250 | 4333.792 | 2048.500 | 3574.000 | 4227.583 | 4113.767 | 1604.017 | 7298.667 | NA |  |
| Q1, Q3 | 4149.917, 8014.167 | 5255.792, 9589.583 | 4704.300, 6752.800 | 9722.667, 13296.667 | 5021.083, 9248.667 | 7535.167, 11648.933 | 5352.483, 6956.500 | 8801.333, 16100.000 | NA |  |
| **Total Cells Group** |  |  |  |  |  |  |  |  |  |  |
| N-Miss | 0 | 0 | 0 | 0 | 0 | 0 | 0 | 0 | 454 | <0.001 (1)/NA |
| <10K | 33 (86.8%) | 5 (83.3%) | 15 (100.0%) | 2 (40.0%) | 7 (87.5%) | 3 (42.9%) | 13 (86.7%) | 18 (40.0%) | 0 |  |
| >=10K | 5 (13.2%) | 1 (16.7%) | 0 (0.0%) | 3 (60.0%) | 1 (12.5%) | 4 (57.1%) | 2 (13.3%) | 27 (60.0%) | 0 |  |
| **Absolute CD4 count** |  |  |  |  |  |  |  |  |  |  |
| N | 38 | 6 | 15 | 5 | 8 | 7 | 15 | 45 | 0 | <0.001 (2)/NA |
| Mean (SD) | 913.920 (481.662) | 486.644 (533.288) | 933.584 (244.616) | 637.592 (339.300) | 1072.958 (696.269) | 636.636 (340.424) | 825.242 (370.458) | 472.843 (430.753) | NA |  |
| Median | 815.192 | 273.719 | 1005.055 | 572.796 | 928.814 | 648.703 | 715.385 | 333.146 | NA |  |
| IQR | 662.490 | 356.040 | 308.368 | 345.786 | 542.497 | 527.786 | 314.327 | 507.811 | NA |  |
| Q1, Q3 | 583.533, 1246.023 | 200.299, 556.340 | 764.717, 1073.085 | 479.042, 824.828 | 653.066, 1195.563 | 337.763, 865.548 | 617.136, 931.463 | 147.000, 654.811 | NA |  |
| **CD4 count group** |  |  |  |  |  |  |  |  |  |  |
| N-Miss | 0 | 0 | 0 | 0 | 0 | 0 | 0 | 0 | 454 | <0.001 (1)/NA |
| <500 | 6 (15.8%) | 4 (66.7%) | 0 (0.0%) | 2 (40.0%) | 2 (25.0%) | 3 (42.9%) | 3 (20.0%) | 31 (68.9%) | 0 |  |
| >=500 | 32 (84.2%) | 2 (33.3%) | 15 (100.0%) | 3 (60.0%) | 6 (75.0%) | 4 (57.1%) | 12 (80.0%) | 14 (31.1%) | 0 |  |
| **CD4 count group** |  |  |  |  |  |  |  |  |  |  |
| N-Miss | 0 | 0 | 0 | 0 | 0 | 0 | 0 | 0 | 454 | <0.001 (1)/NA |
| <200 | 1 (2.6%) | 2 (33.3%) | 0 (0.0%) | 0 (0.0%) | 0 (0.0%) | 0 (0.0%) | 0 (0.0%) | 16 (35.6%) | 0 |  |
| >=200 | 37 (97.4%) | 4 (66.7%) | 15 (100.0%) | 5 (100.0%) | 8 (100.0%) | 7 (100.0%) | 15 (100.0%) | 29 (64.4%) | 0 |  |
| **Absolute CD8 count** |  |  |  |  |  |  |  |  |  |  |
| N | 38 | 6 | 15 | 5 | 8 | 7 | 15 | 45 | 0 | <0.001 (2)/NA |
| Mean (SD) | 503.818 (211.825) | 364.275 (198.376) | 623.986 (253.032) | 575.714 (147.654) | 621.598 (443.857) | 739.736 (559.344) | 509.377 (301.371) | 337.628 (266.247) | NA |  |
| Median | 485.202 | 347.978 | 585.419 | 580.940 | 435.904 | 654.473 | 504.629 | 234.033 | NA |  |
| IQR | 326.397 | 225.972 | 149.542 | 131.185 | 509.178 | 597.697 | 356.402 | 305.954 | NA |  |
| Q1, Q3 | 327.897, 654.293 | 268.263, 494.235 | 505.166, 654.708 | 531.638, 662.823 | 306.542, 815.720 | 351.226, 948.922 | 302.780, 659.182 | 171.639, 477.593 | NA |  |
| **Absolute Bcell count** |  |  |  |  |  |  |  |  |  |  |
| N | 38 | 6 | 15 | 5 | 8 | 7 | 15 | 45 | 0 | 0.058 (2)/NA |
| Mean (SD) | 303.235 (198.682) | 245.900 (196.381) | 332.215 (186.570) | 478.901 (124.642) | 384.034 (280.942) | 604.049 (386.847) | 286.176 (139.569) | 305.913 (188.618) | NA |  |
| Median | 271.938 | 218.719 | 273.015 | 523.123 | 295.603 | 375.512 | 240.171 | 288.146 | NA |  |
| IQR | 149.371 | 168.348 | 110.418 | 193.315 | 222.009 | 621.408 | 197.012 | 200.188 | NA |  |
| Q1, Q3 | 184.956, 334.327 | 109.076, 277.424 | 237.301, 347.719 | 379.731, 573.046 | 217.026, 439.036 | 286.432, 907.839 | 186.353, 383.364 | 173.700, 373.888 | NA |  |
| **Absolute Natural Killer cell count** |  |  |  |  |  |  |  |  |  |  |
| N | 38 | 6 | 15 | 5 | 8 | 7 | 15 | 45 | 0 | 0.139 (2)/NA |
| Mean (SD) | 354.781 (180.399) | 270.880 (203.816) | 356.665 (180.926) | 319.187 (230.643) | 285.684 (187.400) | 448.519 (539.831) | 363.965 (189.277) | 264.217 (127.918) | NA |  |
| Median | 346.911 | 237.071 | 316.476 | 193.064 | 198.822 | 247.054 | 358.295 | 232.539 | NA |  |
| IQR | 165.185 | 222.523 | 216.362 | 272.796 | 113.257 | 136.726 | 165.078 | 113.562 | NA |  |
| Q1, Q3 | 244.128, 409.313 | 121.730, 344.252 | 227.197, 443.560 | 162.758, 435.554 | 186.936, 300.194 | 211.686, 348.412 | 245.508, 410.587 | 195.477, 309.039 | NA |  |
| **Absolute Lymphocyte count** |  |  |  |  |  |  |  |  |  |  |
| N | 38 | 6 | 15 | 5 | 8 | 7 | 15 | 45 | 0 | <0.001 (2)/NA |
| Mean (SD) | 2075.753 (816.808) | 1367.699 (922.176) | 2246.450 (715.832) | 2011.394 (668.872) | 2364.274 (1389.322) | 2428.941 (1005.193) | 1984.760 (686.555) | 1380.601 (874.485) | NA |  |
| Median | 2015.744 | 1078.599 | 2245.097 | 1907.975 | 1963.948 | 2380.005 | 1976.597 | 1112.395 | NA |  |
| IQR | 1232.301 | 760.034 | 718.506 | 820.224 | 1149.677 | 933.993 | 828.516 | 967.532 | NA |  |
| Q1, Q3 | 1393.659, 2625.961 | 910.874, 1670.909 | 1700.856, 2419.362 | 1809.826, 2630.050 | 1534.964, 2684.641 | 1953.389, 2887.382 | 1466.608, 2295.124 | 752.700, 1720.232 | NA |  |
| **Absolute Lymphocyte Count group** |  |  |  |  |  |  |  |  |  |  |
| N-Miss | 0 | 0 | 0 | 0 | 0 | 0 | 0 | 0 | 454 | <0.001 (1)/NA |
| <1K | 3 (7.9%) | 3 (50.0%) | 0 (0.0%) | 0 (0.0%) | 0 (0.0%) | 1 (14.3%) | 1 (6.7%) | 20 (44.4%) | 0 |  |
| >=1K | 35 (92.1%) | 3 (50.0%) | 15 (100.0%) | 5 (100.0%) | 8 (100.0%) | 6 (85.7%) | 14 (93.3%) | 25 (55.6%) | 0 |  |
| **Absolute Neutrophil count** |  |  |  |  |  |  |  |  |  |  |
| N | 38 | 6 | 15 | 5 | 8 | 7 | 15 | 45 | 0 | <0.001 (2)/NA |
| Mean (SD) | 4046.144 (1924.970) | 5211.272 (2853.650) | 3323.085 (1362.543) | 9833.801 (3227.779) | 4595.149 (2378.296) | 7195.398 (1914.126) | 3876.212 (2331.578) | 10636.960 (4351.811) | NA |  |
| Median | 3602.798 | 5150.332 | 3231.922 | 10451.653 | 3671.150 | 7289.448 | 2973.899 | 10032.306 | NA |  |
| IQR | 2542.651 | 4346.295 | 2010.010 | 4416.693 | 2339.802 | 2591.905 | 1965.257 | 5813.876 | NA |  |
| Q1, Q3 | 2471.387, 5014.039 | 3428.589, 7774.884 | 2268.662, 4278.672 | 6960.063, 11376.756 | 3137.749, 5477.551 | 5763.777, 8355.682 | 2400.525, 4365.782 | 7440.824, 13254.700 | NA |  |
| **Absolute Neutrophil count group** |  |  |  |  |  |  |  |  |  |  |
| N-Miss | 0 | 0 | 0 | 0 | 0 | 0 | 0 | 0 | 454 | <0.001 (1)/NA |
| <8870 | 37 (97.4%) | 6 (100.0%) | 15 (100.0%) | 2 (40.0%) | 7 (87.5%) | 6 (85.7%) | 14 (93.3%) | 19 (42.2%) | 0 |  |
| >=8870 | 1 (2.6%) | 0 (0.0%) | 0 (0.0%) | 3 (60.0%) | 1 (12.5%) | 1 (14.3%) | 1 (6.7%) | 26 (57.8%) | 0 |  |
| **Absolute Monocyte count** |  |  |  |  |  |  |  |  |  |  |
| N | 38 | 6 | 15 | 5 | 8 | 7 | 15 | 45 | 0 | 0.505 (2)/NA |
| Mean (SD) | 471.712 (190.555) | 396.035 (275.135) | 406.765 (115.793) | 800.182 (354.955) | 587.346 (344.356) | 500.184 (235.234) | 464.521 (135.581) | 548.180 (354.920) | NA |  |
| Median | 455.490 | 395.010 | 415.355 | 837.876 | 450.692 | 539.862 | 472.409 | 402.332 | NA |  |
| IQR | 215.746 | 454.792 | 102.471 | 541.691 | 226.676 | 173.636 | 180.240 | 468.213 | NA |  |
| Q1, Q3 | 346.519, 562.265 | 151.862, 606.654 | 364.832, 467.302 | 492.268, 1033.959 | 383.229, 609.905 | 389.108, 562.744 | 359.010, 539.250 | 292.093, 760.306 | NA |  |
| **CD4/CD8 ratio** |  |  |  |  |  |  |  |  |  | 0.076/0.004 |
| N | 38 | 6 | 15 | 5 | 8 | 7 | 15 | 45 | 454 |  |
| Mean (SD) | 2.115 (2.133) | 1.222 (0.925) | 1.614 (0.468) | 1.065 (0.433) | 1.932 (0.969) | 1.269 (0.743) | 2.134 (1.717) | 1.518 (1.042) | 2.186 (1.844) |  |
| Median | 1.793 | 1.018 | 1.533 | 0.986 | 1.601 | 1.299 | 1.421 | 1.179 | 1.746 |  |
| IQR | 0.981 | 0.776 | 0.671 | 0.750 | 0.454 | 0.844 | 1.455 | 1.015 | 1.360 |  |
| Q1, Q3 | 1.226, 2.207 | 0.649, 1.425 | 1.335, 2.006 | 0.723, 1.473 | 1.438, 1.893 | 0.870, 1.714 | 1.154, 2.609 | 0.751, 1.766 | 1.209, 2.569 |  |
| **Neutrophil/Lymphocyte Ratio** |  |  |  |  |  |  |  |  |  | <0.001/< 0.001 |
| N | 38 | 6 | 15 | 5 | 8 | 7 | 15 | 45 | 454 |  |
| Mean (SD) | 2.106 (1.184) | 4.713 (3.162) | 1.527 (0.656) | 5.528 (2.918) | 2.043 (0.405) | 3.411 (1.722) | 2.273 (1.834) | 10.184 (6.053) | 1.841 (1.140) |  |
| Median | 1.784 | 3.352 | 1.379 | 5.357 | 1.980 | 3.025 | 1.471 | 9.883 | 1.572 |  |
| IQR | 1.085 | 4.694 | 0.454 | 2.638 | 0.497 | 0.910 | 1.286 | 9.967 | 1.054 |  |
| Q1, Q3 | 1.443, 2.528 | 2.449, 7.143 | 1.122, 1.576 | 3.648, 6.286 | 1.797, 2.294 | 2.537, 3.448 | 1.192, 2.478 | 4.799, 14.766 | 1.159, 2.213 |  |
| **Neutrophil/Lymphocyte Ratio grouped** |  |  |  |  |  |  |  |  |  | <0.001/< 0.001 |
| <4 | 37 (97.4%) | 4 (66.7%) | 15 (100.0%) | 2 (40.0%) | 8 (100.0%) | 6 (85.7%) | 13 (86.7%) | 6 (13.3%) | 438 (96.5%) |  |
| ≥4 | 1 (2.6%) | 2 (33.3%) | 0 (0.0%) | 3 (60.0%) | 0 (0.0%) | 1 (14.3%) | 2 (13.3%) | 39 (86.7%) | 16 (3.5%) |  |
| **Lymphocyte/Monocyte Ratio** |  |  |  |  |  |  |  |  |  |  |
| N | 38 | 6 | 15 | 5 | 8 | 7 | 15 | 45 | 454 | <0.001/<0.001 |
| Mean (SD) | 4.595 (1.257) | 4.534 (2.681) | 5.928 (2.286) | 2.946 (1.403) | 4.264 (1.750) | 5.179 (1.418) | 4.464 (1.472) | 3.046 (1.750) | 5.187 (2.155) |  |
| Median | 4.601 | 4.670 | 5.644 | 3.178 | 4.099 | 4.608 | 4.693 | 2.619 | 4.793 |  |
| IQR | 1.333 | 4.266 | 1.941 | 1.752 | 2.012 | 2.002 | 2.626 | 2.045 | 2.610 |  |
| Q1, Q3 | 3.874, 5.207 | 2.295, 6.561 | 4.662, 6.603 | 2.124, 3.876 | 2.957, 4.969 | 4.150, 6.152 | 3.351, 5.977 | 1.694, 3.738 | 3.754, 6.363 |  |
| **Total lymphocyte %** |  |  |  |  |  |  |  |  |  | <0.001/< 0.001 |
| N | 38 | 6 | 15 | 5 | 8 | 7 | 15 | 45 | 454 |  |
| Mean (SD) | 0.329 (0.082) | 0.209 (0.096) | 0.391 (0.078) | 0.170 (0.073) | 0.311 (0.047) | 0.239 (0.067) | 0.344 (0.124) | 0.118 (0.067) | 0.371 (0.109) |  |
| Median | 0.334 | 0.214 | 0.393 | 0.149 | 0.316 | 0.235 | 0.365 | 0.090 | 0.371 |  |
| IQR | 0.120 | 0.153 | 0.074 | 0.071 | 0.058 | 0.051 | 0.153 | 0.104 | 0.145 |  |
| Q1, Q3 | 0.265, 0.384 | 0.126, 0.278 | 0.370, 0.444 | 0.136, 0.207 | 0.277, 0.335 | 0.219, 0.270 | 0.273, 0.425 | 0.063, 0.166 | 0.294, 0.439 |  |

1. Pearson’s Chi-squared test

2. Kruskal-Wallis rank sum test

*p-value for analyses using cases only

** p-values for analyses including IPS glioma patients and AGS controls

**Supplemental Table 7:** Characteristics of Dexamethasone use at Blood draw by Glioma WHO 2016 Classification

|  | IDH MUT 1p19q codel oligo (N=44) | IDH MUT Astro (N=20) | IDH MUT GBM (N=15) | IDH WT GBM (N=60) | p value |
| --- | --- | --- | --- | --- | --- |
| **Dexamethasone use in past mo** |  |  |  |  | < 0.001 |
| No | 38 (86.4%) | 14 (70.0%) | 7 (46.7%) | 9 (15.0%) |  |
| Yes | 6 (13.6%) | 6 (30.0%) | 8 (53.3%) | 51 (85.0%) |  |
| **Dexamethasone at blood draw** |  |  |  |  | < 0.001 |
| No | 38 (86.4%) | 15 (75.0%) | 8 (53.3%) | 15 (25.0%) |  |
| Yes | 6 (13.6%) | 5 (25.0%) | 7 (46.7%) | 45 (75.0%) |  |
| **Cumulative dexamethasone use in mg** |  |  |  |  | 0.358 |
| N | 6 | 6 | 8 | 51 |  |
| Mean (SD) | 45.500 (43.58) | 108.250 (83.87) | 47.812 (33.91) | 82.049 (83.90) |  |
| Median | 25.000 | 75.000 | 42.000 | 56.000 |  |
| Q1, Q3 | 13.500, 71.000 | 52.750, 173.750 | 22.500, 75.375 | 23.000, 114.000 |  |
| **Cumulative dexamethasone among users at blood draw** |  |  |  |  | 0.183 |
| N | 6 | 5 | 7 | 45 |  |
| Mean (SD) | 45.500 (43.58) | 125.200 (81.48) | 50.929 (35.37) | 89.711 (86.07) |  |
| Median | 25.00 | 95.00 | 56.00 | 60.00 |  |
| Q1, Q3 | 13.50, 71.00 | 55.00, 200.00 | 20.00, 80.25 | 32.00, 124.00 |  |
| **Dexamethasone mg/dose** |  |  |  |  | 0.210 |
| N | 6 | 6 | 8 | 51 |  |
| Mean (SD) | 2.833 (1.329) | 2.417 (1.357) | 3.062 (3.099) | 3.373 (1.367) |  |
| Median | 3.000 | 2.000 | 2.000 | 4.000 |  |
| Q1, Q3 | 2.000, 4.000 | 2.000, 3.500 | 1.000, 4.000 | 2.000, 4.000 |  |
| **Dexamethasone mg/dose among users at blood draw** |  |  |  |  | 0.274 |
| N | 6 | 5 | 7 | 45 |  |
| Mean (SD) | 2.833 (1.329) | 2.800 (1.095) | 3.357 (3.224) | 3.467 (1.036) |  |
| Median | 3.000 | 2.000 | 2.000 | 4.000 |  |
| Q1, Q3 | 2.000, 4.000 | 2.000, 4.000 | 1.500, 4.000 | 3.000, 4.000 |  |
| **Dexamethasone doses/day** |  |  |  |  | 0.010 |
| N | 6 | 6 | 8 | 51 |  |
| Mean (SD) | 2.667 (0.816) | 2.167 (0.753) | 1.625 (0.518) | 2.765 (0.971) |  |
| Median | 2.500 | 2.000 | 2.000 | 3.000 |  |
| Q1, Q3 | 2.000, 3.000 | 2.000, 2.750 | 1.000, 2.000 | 2.000, 3.500 |  |
| **Dexamethasone doses/day, among users at blood draw** |  |  |  |  | 0.004 |
| N | 6 | 5 | 7 | 45 |  |
| Mean (SD) | 2.667 (0.816) | 2.400 (0.548) | 1.714 (0.488) | 2.956 (0.824) |  |
| Median | 2.500 | 2.000 | 2.000 | 3.000 |  |
| Q1, Q3 | 2.000, 3.000 | 2.000, 3.000 | 1.500, 2.000 | 2.000, 4.000 |  |
| **Total days on dexamethasone** |  |  |  |  | 0.221 |
| N | 6 | 6 | 8 | 51 |  |
| Mean (SD) | 10.667 (12.209) | 13.500 (5.958) | 9.625 (4.926) | 9.000 (8.052) |  |
| Median | 5.500 | 13.000 | 8.500 | 6.000 |  |
| Q1, Q3 | 1.250, 18.000 | 9.000, 16.250 | 7.000, 11.000 | 3.500, 12.500 |  |
| **Total days on dexamethasone, among users at blood draw** |  |  |  |  | 0.302 |
| N | 6 | 5 | 7 | 45 |  |
| Mean (SD) | 10.667 (12.209) | 14.600 (5.941) | 9.714 (5.314) | 9.444 (8.363) |  |
| Median | 5.500 | 14.000 | 8.000 | 6.000 |  |
| Q1, Q3 | 1.250, 18.000 | 12.000, 17.000 | 7.000, 13.000 | 4.000, 14.000 |  |
| **Dexamethasone/day, among users at blood draw** |  |  |  |  | 0.080 |
| N | 6 | 5 | 7 | 45 |  |
| Mean (SD) | 8.306 (5.741) | 9.100 (5.097) | 5.223 (2.959) | 10.345 (4.896) |  |
| Median | 8.000 | 7.917 | 4.147 | 12.000 |  |
| Q1, Q3 | 3.875, 12.000 | 7.429, 11.765 | 4.000, 6.706 | 6.000, 13.778 |  |

1. Pearson’s Chi-squared test for categorical factors

2. Kruskal-Wallis rank sum test for continuous factors

**Supplemental Table 8**: Association of Older Age and Abnormal Immune Profiles in Glioma Patients stratified by Dexamethasone Use and Glioma Grade

|  | **No DEX** | | | | |  | **DEX** | | | | |  |
| --- | --- | --- | --- | --- | --- | --- | --- | --- | --- | --- | --- | --- |
|  | **Grade II-III** | |  | **Grade IV** | |  | **Grade II-III** | |  | **Grade IV** | |  |
|  | **Age <58** | **Age >=58** |  | **Age <58** | **Age >=58** |  | **Age <58** | **Age >=58** |  | **Age <58** | **Age >=58** |  |
|  | **n (%)** | **n (%)** |  | **n (%)** | **n (%)** |  | **n (%)** | **n (%)** |  | **n (%)** | **n (%)** |  |
| **CD4 count <500** | 5 (10) | 1 (20) |  | 3(27) | 2 (17) |  | 5 (56) | 1 (50) |  | 8 (44) | 26 (77) |  |
| **CD4 count >=500** | 43 (90) | 4 (80) |  | 8 (73) | 10 (83) |  | 4 (44) | 1 (50) |  | 10 (56) | 8 (24) |  |
| **Fisher's Exact p value** |  | 0.46 |  |  | 0.64 |  |  | 1 |  |  | **0.03** |  |
| **Mantel Haenszel p-value** |  |  |  |  |  |  |  |  |  |  |  | 0.1 |
| **Breslow-Day test for homogeneity** |  |  |  |  |  |  |  |  |  |  |  | 0.33 |
|  |  |  |  |  |  |  |  |  |  |  |  |  |
| **CD4 count <200** | 1 (2) | 0 |  | 0 | 0 |  | 2 (22) | 0 |  | 1 (6) | 15 (44) |  |
| **CD4 count >=200** | 47 (98) | 5 (100) |  | 11 (100) | 12 (100) |  | 7 (78) | 2 (100) |  | 17 (94) | 19 (56) |  |
| **Fisher's Exact p value** |  | 1 |  |  |  |  |  | 1 |  |  | **0.004** |  |
| **Mantel Haenszel p-value** |  |  |  |  |  |  |  |  |  |  |  | 0.02 |
| **Breslow-Day test for homogeneity** |  |  |  |  |  |  |  |  |  |  |  | 0.16 |
|  |  |  |  |  |  |  |  |  |  |  |  |  |
| **Absolute Neutrophil Count <8870** | 48 (100) | 4 (80) |  | 9 (82) | 12 (100) |  | 6 (67) | 2 (100) |  | 13 (72) | 12 (35) |  |
| **Absolute Neutrophil Count >=8870** | 0 | 1 (20) |  | 2 (18) | 0 |  | 3 (33) | 0 |  | 5 (28) | 22 (65) |  |
| **Fisher's Exact p value** |  | 0.09 |  |  | 0.22 |  |  | 1 |  |  | **0.02** |  |
| **Mantel Haenszel p-value** |  |  |  |  |  |  |  |  |  |  |  | 0.06 |
| **Breslow-Day test for homogeneity** |  |  |  |  |  |  |  |  |  |  |  | 0.005 |
|  |  |  |  |  |  |  |  |  |  |  |  |  |
| **Absolute Lymphocyte Count <1K** | 3 (6) | 0 |  | 0 | 1 (8) |  | 2 (22) | 1 (50) |  | 2 (11) | 19 (56) |  |
| **Absolute Lymphocyte Count >=1K** | 45 (94) | 5 (100) |  | 11 (100) | 11 (92) |  | 7 (78) | 1 (50) |  | 16 (89) | 15 (44) |  |
| **Fisher's Exact p value** |  | 1 |  |  | 1 |  |  | 0.49 |  |  | **0.002** |  |
| **Mantel Haenszel p-value** |  |  |  |  |  |  |  |  |  |  |  | 0.002 |
| **Breslow-Day test for homogeneity** |  |  |  |  |  |  |  |  |  |  |  | 0.46 |
|  |  |  |  |  |  |  |  |  |  |  |  |  |
| **Total Cells >=10K** | 44 (92) | 4 (80) |  | 9 (82) | 11 (92) |  | 5 (56) | 2 (100) |  | 9 (50) | 12 (35) |  |
| **Total Cells <10K** | 4 (8) | 1 (20) |  | 2 918) | 1 (8) |  | 4 (44) | 0 |  | 9 (50) | 22 (65) |  |
| **Fisher's Exact p value** |  | 0.4 |  |  | 0.59 |  |  | 0.49 |  |  | 0.38 |  |
| **Mantel Haenszel p-value** |  |  |  |  |  |  |  |  |  |  |  | 0.64 |
| **Breslow-Day test for homogeneity** |  |  |  |  |  |  |  |  |  |  |  | 0.34 |
|  |  |  |  |  |  |  |  |  |  |  |  |  |
| **Neutrophil/Lymphocyte Ratio <4** | 47 (98) | 5 (100) |  | 10 (91) | 11 (92) |  | 5 (56) | 1 (50) |  | 11 (61) | 1 (3) |  |
| **Neutrophil/Lymphocyte Ratio >=4** | 1 (2) | 0 |  | 1 (9) | 1 (8) |  | 4 (44) | 1 (50) |  | 7 (39) | 33 (97) |  |
| **Fisher's Exact p value** |  | 1 |  |  | 1 |  |  | 1 |  |  | <0.0001 |  |
| **Mantel Haenszel p-value** |  |  |  |  |  |  |  |  |  |  |  | 0.0001 |
| **Breslow-Day test for homogeneity** |  |  |  |  |  |  |  |  |  |  |  | 0.04 |
